# Myocardial and Vascular Involvement in COVID-19 and Post-Vaccination States: Understanding Injury Pathways and Clinical Implications

**DOI:** 10.3390/life16020268

**Published:** 2026-02-04

**Authors:** Roxana-Nicoleta Siliste, Serban Benea, Corina Homentcovschi, Teodora Deaconu, Constantin Caruntu, Ilinca Savulescu-Fiedler

**Affiliations:** 1 Department of Internal Medicine, Coltea Clinical Hospital, 030167 Bucharest, Romania; roxana.siliste@umfcd.ro (R.-N.S.); ilinca.savulescu@umfcd.ro (I.S.-F.); 2 Department of Internal Medicine, “Carol Davila” University of Medicine and Pharmacy, 050474 Bucharest, Romania; 3 National Institute for Infectious Diseases “Prof. Dr. Matei Bals”, 021105 Bucharest, Romania; gadeateodora@yahoo.com; 4 Department of Infectious Diseases, “Carol Davila” University of Medicine and Pharmacy, 050474 Bucharest, Romania; 5 Department of Dermatology, “Prof. N.C. Paulescu” National Institute of Diabetes, Nutrition and Metabolic Diseases, 011233 Bucharest, Romania; constantin.caruntu@umfcd.ro; 6 Department of Physiology, “Carol Davila” University of Medicine and Pharmacy, 050474 Bucharest, Romania

**Keywords:** SARS-CoV-2, myocarditis, endothelial dysfunction, cardiovascular injury, COVID-19 vaccination, mRNA vaccines, immune-mediated inflammation, cytokine storm, oxidative stress, ACE2 receptor

## Abstract

Myocardial and vascular injury secondary to SARS-CoV-2 infection and vaccination has emerged as a clinically relevant phenomenon, with distinct but overlapping mechanisms. Myocardial injury in COVID-19 results from a complex interplay between direct viral effects and immune-mediated inflammation, supported by histopathological studies revealing macrophage-rich infiltrates, microthrombosis, and supporting fibrosis in isolated areas. In contrast, vaccine-associated myocarditis—reported predominantly following mRNA vaccines—has a self-limiting clinical course, with mechanisms likely involving molecular mimicry, aberrant immune activation, or hypersensitivity reactions, although these pathways require further validation. Although mRNA vaccines have been associated with a small increase in myocarditis, particularly in young men, the risk is significantly lower than that associated with COVID-19 infection, and the cardiovascular benefits of vaccination far outweigh these rare adverse events in most populations. After the end of the pandemic, the number of patients with severe forms of COVID-19 has decreased significantly, but we consider that cardiac involvement remains an important issue for the acute and long-term prognosis of patients with SARS-CoV-2 infection. Our paper synthesizes the latest epidemiological and mechanistic evidence on the link between COVID-19, vaccination, and myocardial and/or vascular injuries, highlighting the clinical implications and providing practical recommendations for management, as well as future perspectives on risk assessment, targeted immunotherapy, advanced diagnostic tools, and long-term monitoring.

## 1. Introduction

With the emergence of the COVID-19 pandemic, SARS-CoV-2 has demonstrated clear potential for cardiovascular involvement, with myocarditis increasingly being recognized as one of the most common inflammatory cardiac manifestations associated with both natural infection and, to a much lesser extent, vaccination against COVID-19 [1,2,3]. Clinical studies and post-authorization surveillance systems have documented an increased risk of myocardial injury following SARS-CoV-2 infection, reflecting mechanisms that include direct viral invasion, immune dysregulation, and microvascular and thrombotic imbalances [1,2,3,4]. The incidence of both SARS-CoV-2-related and vaccine-associated myocarditis is higher than that reported in pre-COVID-19 viral infections. Myocardial involvement in COVID-19 is often patchy, and unlike other viral myocarditis—where lymphocytic infiltration is common—in most cases of SARS-CoV-2 myocarditis, lymphocytic invasion is minimal or absent, making myocarditis an immune-mediated condition rather than a result of direct viral cell damage [4,5]. The initial step involves the virus binding to the host cell membrane via the angiotensin-converting enzyme 2 (ACE2) receptor, followed by cellular entry. ACE2 is highly expressed in vascular endothelial cells and perivascular mural cells (pericytes), whereas its expression in cardiomyocytes is comparatively lower [6].

Acute SARS-CoV-2 infection has been linked not only to myocarditis but also to acute myocardial infarction caused by atherothrombotic plaque disruption, as well as to infarction resulting from an imbalance between myocardial oxygen supply and demand. It can also cause myocardial injury secondary to multisystem inflammatory syndrome, new-onset or decompensated heart failure in patients with preexisting cardiovascular disease, pulmonary embolism, and a wide range of arrhythmias. These findings highlight the complex cardiovascular burden of COVID-19, which goes beyond myocarditis and emphasizes the need for comprehensive cardiac evaluation and follow-up in affected patients [7,8].

Meanwhile, global vaccination efforts using mRNA-based platforms have been linked to a small but measurable rise in myocarditis cases, usually occurring within the first week after vaccination, more often after the second dose, and mainly affecting young male recipients [2]. The Israeli Ministry of Health reported notably lower rates of myocarditis after the third dose compared to the second dose, regardless of age or gender [9]. Nevertheless, the absolute risk of myocarditis after vaccination remains low compared to the much higher incidence of COVID-19-related cardiac involvement, which is estimated to be up to 100 times greater [3].

This paper aims to synthesize the latest epidemiological and mechanistic evidence on myocardial and vascular injury in SARS-CoV-2 infection, as well as on vaccination-associated myocarditis; to explore proposed pathogenic pathways and clinical implications; to provide practical recommendations for assessment, management, and monitoring; and, last but not least, provide future perspectives on risk assessment, targeted immunotherapy, advanced diagnostic tools, and long-term monitoring.

## 2. Methodology

This narrative review synthesizes current evidence on cardiovascular involvement in SARS-CoV-2 infection and following vaccination with emphasis on myocarditis. Our team conducted extensive literature research across major electronic databases such as PubMed, Google Scholar, and Embase. The search strategy used a combination of key terms like “viral myocarditis”, “endothelial dysfunction”, “cardiovascular injury”, “mRNA vaccines”, “immune-mediated inflammation”, “cytokine storm”, and “oxidative stress” alongside “COVID-19” and “SARS-CoV-2 vaccination”. Articles were included if they (1) were published in English; (2) were available in full text; (3) addressed cardiovascular complications of COVID-19 infection or vaccination like myocardial injury, myocarditis, endothelial dysfunction, microvascular thrombosis; (4) included original research, systematic reviews, meta-analyses, case series, or clinical guidelines. In addition, reference lists of key articles and relevant reviews were screened to identify further eligible studies. Priority was given to large population-based studies, meta-analyses, and high-quality cohort studies, while mechanistic hypotheses were interpreted considering the overall strength and consistency of the evidence.

Additionally, our team reviewed grey literature sources to ensure a broad and unbiased overview of the topic and include studies that might not be indexed in major databases.

Evidence was synthesized qualitatively. Given the narrative design, we did not perform a formal risk-of-bias assessment or meta-analysis; instead, we integrated findings by study type and topic to highlight areas of consistency, uncertainty, and research priorities.

## 3. Epidemiology of Myocardial Involvement in COVID-19

Myocarditis, defined as inflammation of the myocardium caused by infectious agents or non-infectious processes, has an estimated global prevalence of 10.2–105.6 cases per 100,000 people annually [10], with a higher prevalence in males [11]. Viral infections account for 69% of myocarditis cases [11], and 1–5% of patients with viral infections develop myocarditis [12].

The incidence of myocarditis before COVID-19 was lower than the incidence reported during COVID-19. The incidence of myocarditis pre-COVID was 1 to 10 cases per 100,000 individuals [13], whereas during SARS-CoV-2 infection, it ranged from 150 to 4000 cases per 100,000 individuals [13,14].

The incidence of myocarditis linked to COVID-19 is estimated to be about 450 cases per million young adults, although the true rate remains unknown [15]. It has been diagnosed in 2 to 4 patients per 1000 hospitalizations [16]. Myocardial injury, defined as an increase in troponin levels, is more common among patients with SARS-CoV-2 infection, with a prevalence of 22% among all hospitalized patients, rising to nearly 30% in elderly patients or those with severe disease [17]. The incidence of acute myocardial infarction (AMI) in patients with SARS-CoV-2 varies depending on the studied population. A systematic review and meta-analysis of over 1.2 million COVID-19 survivors found an incidence of 3.5 cases per 1000 people (0.35%) compared to 2.02 cases per 1000 patients (0.2%) in the control group [18]. The incidence of AMI in hospitalized patients ranges from 1.1% to 8.9% [19]. In a large U.S. surveillance study, the incidence was 5.5% [20].

## 4. Histology of COVID-19 Myocarditis

Generally, the typical histological changes of viral myocarditis include infiltrates of lymphocytes, neutrophils, monocytes, histiocytes, eosinophils, or giant cells, along with vasculitis, necrosis, and myocardial fibrosis [11]. Fulminant myocarditis is characterized by multiple areas of active inflammation and myocyte necrosis [11].

Histopathological analyses of myocardial tissue from patients with confirmed SARS-CoV-2 infection have revealed a pattern that differs substantially from that of classical viral myocarditis. In contrast to enteroviral or adenoviral myocarditis, which are characterized by diffuse lymphocytic infiltration and direct myocyte necrosis, most COVID-19 autopsy studies demonstrate minimal or focal inflammatory infiltrates, often insufficient to fulfill Dallas criteria for active myocarditis [5,21]. Myocarditis, even when observed, tends to be patchy and usually occurs without the dense mononuclear infiltrates typically seen in conventional viral myocarditis, indicating the possibility that direct cytopathic injury is not the main mechanism [22].

Autopsies of patients with severe COVID-19 revealed significant arterial vascular changes in various organs, such as hemorrhage and endothelitis [23,24,25], endothelial swelling, microvascular rarefaction [4] associated with thrombosis, and inflammation [5,26]. Thrombotic microangiopathy, a result of endothelial dysfunction, underlies many ischemic events, as well as heart failure and renal dysfunction [27]. A consistent histological feature reported across multiple cohorts is extensive microvascular and endothelial involvement. SARS-CoV-2 demonstrates tropism for endothelial cells and pericytes due to high ACE2 expression [4]. Numerous studies have described capillary congestion, endothelial activation, microthrombi, fibrin deposits, and complement-mediated lesions. All of these findings support a model of microcirculatory dysfunction rather than primary myocyte invasion. These vascular abnormalities are thought to contribute to myocardial ischemia, regional injury, and the patchy necrotic lesions observed in most histological specimens [4,28].

The most common histologic pattern in COVID-19 myocarditis is described as a cellular infiltrate, mainly consisting of macrophages (CD68) and granulomas (giant cells and eosinophils) [26]. Most cases of SARS-CoV-2 myocarditis lack lymphocytic invasion; even near necrotic myocytes, typical lymphocytic infiltrates are not observed [28]. Some autopsy studies show limited infiltrates of T-cells (CD4) without significant CD8+ cells or B-cells (CD20) infiltration [29]. Multiple studies have shown mild interstitial edema without clear myocyte destruction [5,30].

The myocardial fibrosis in COVID-19 is a controversial topic; some authors have reported only limited interstitial fibrosis [29].

One study [26] on individuals who died with COVID-19 showed viral replication within the heart, a direct correlation between the myocardial density of macrophages and lymphocytes, a direct relationship between the presence of SARS-CoV-2 infected myocytes and myocarditis, and a connection between apparent SARS-CoV-2 infected endothelial cells and thrombosis. Other autopsy reports indicate that the percentage of SARS-CoV-2 evidence in the hearts of deceased individuals was high (62%), without extensive cell infiltrates or necrosis within the myocardium (V. Coronaviridae Study Group of the International Committee on Taxonomy of the species Severe acute respiratory syndrome-related coronavirus). Most studies have found that the detection of SARS-CoV-2 RNA or proteins within myocardial cells is variable and often low, further supporting the idea that immune-vascular mechanisms play a key role in COVID-19 myocardial pathology [21,30,31,32].

The inconsistency in lymphocytic infiltration, along with the prominence of macrophage infiltration and microvascular lesions, supports the idea that COVID-19-related myocardial damage results from systemic immune dysregulation, cytokine-driven injury, and endothelial dysfunction rather than direct viral cytotoxicity [33].

## 5. Myocarditis Pathophysiology

It is not yet clear whether direct viral intervention or autoimmunity is the main determinant of viral myocarditis. After entry into the myocytes, the virus is eliminated through macrophage and lymphocyte intervention, or, if the viral genome persists, inflammation and the autoimmune response cause myocardial damage [34,35].

The pathogenesis of viral myocarditis is explained as a three-phase model.

The acute phase, characterized by high virus levels, lasts 1–7 days and is represented by acute cardiac cell damage and cellular death, secondary to the innate immune response [36]. The presence of the virus within the heart tissue triggers immune system activation, the production of inflammatory mediators and cardiac auto-antibodies [37], activation of macrophages, and the accumulation of mast cells, natural killer T cells, and neutrophils in the myocardium [38,39].

The viruses multiply within the cardiomyocytes, causing cytoskeletal breakdown, which leads to myocardial damage [40], and also results in the release of myocyte antigens, IL-1β, pathogen-associated molecular patterns (PAMPs), and damage-associated molecular patterns (DAMPs) into the bloodstream [37]. IL-1β, PAMPs, and DAMPs promote extramedullary hematopoiesis, including the release of pro-inflammatory Ly6Chigh monocytes [41], precursors to macrophages, shown in mice models. The cardiomyocytes produce interferon gamma (IFNy), stimulating fibroblasts to produce chemokines and chemoattractants for Ly6Chigh monocytes [42]. Monocyte recruitment is a double-edged sword—effective for removing the virus from the myocardium, but also as a trigger for the loss of myocyte contractile units [42].

Mast cells and neutrophils (PMNs) migrate within the myocardium as early events in viral infections [11], leading to different outcomes. Mast cells produce cytokines with antiviral effects (such as TNFα, IL-1β, and IL-4) [40], enzymes acting as fibroblast inductors, such as matrix metalloproteinases, fibrinogenic mediators like chymases and tryptases, which offer protective effects against myocardial fibrosis and necrosis [43]. PMNs are involved in both initiating and sustaining inflammation, forming extracellular neutrophil traps [44]. Their recruitment is followed by the release of pro-inflammatory mediators (myeloperoxidase, IL-6, IL-8, TNFα, alarmins) and adhesion to fibrinogen and fibronectin [40,45]. Furthermore, PMNs promote the migration of CD8+ T lymphocytes to the affected myocardium, contributing to myocarditis progression [46].

**Figure 1:**
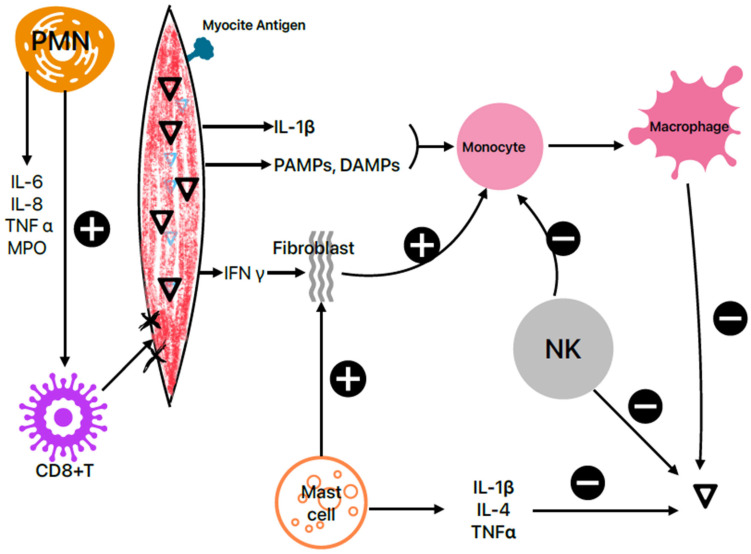
The acute phase of viral myocarditis. Mast cells and neutrophil (PMN) migration within the myocardium is observed earlier in viral infections, with different consequences on inflammation; the cytokines synthesized by mast cells have an antiviral action, meanwhile, the cytokines secreted by PMNs have proinflammatory effects. The natural killer cells (NK) limit both viral replication and inflammatory responses. The virus multiplies within cardiomyocytes, leading to the release of CD14+CD16-monocytes, precursors of macrophages. PAMPs—pathogen-associated molecular patterns, DAMPs—damage-associated molecular patterns; TNFα—α tumor necrosis factor; IFNγ—γ interferon; ▽—virus.

Besides mast cells, natural killer (NK) cells [11] also play protective roles. NKs are involved in clearing viral infections and limiting both viral replication and inflammatory responses [47]. NKs also suppress monocyte maturation [42] (Figure 1).

The subacute immune phase lasts from 1 to 4 weeks, with CD4+ T cells playing a key role [36] in the adaptive immune response. During this phase, viral titers are lower than in the earlier phase [11]. Activated Th1 cells release various inflammatory markers, such as IL-1α, IL-1β, and TNF-α, which contribute to the destruction of contractile units and the interstitial matrix of infected myocytes [37,48]. Myocyte secretion of IL-1 and TNFα stimulates macrophages to produce IL-1β, with harmful effects on cardiomyocytes [49].

**Figure 2:**
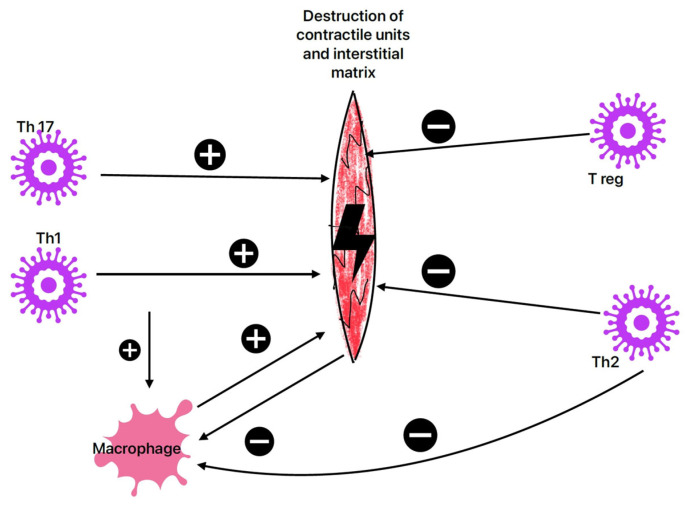
The subacute phase of viral myocarditis. The T cell populations have different effects on cardiomyocytes; T reg and Th2 prevent the destruction of contractile units and interstitial matrix, meanwhile Th17 and Th1 populations have deleterious effects on cardiomyocytes.

Regulatory T-cells (Treg), through their secretion of inhibitory cytokines (TGF-β1 and IL-10) or by expressing surface molecules such as the glucocorticoid-induced TNF receptor, lead to a decrease in inflammation and provide protective effects on cardiac myocytes [50]. Th17 levels correlate with inflammation and the likelihood of myocarditis, dilated cardiomyopathy, and heart failure [51]. Th2 cells produce IL-10 and IL-13, which have anti-inflammatory properties, inhibiting macrophage function and cytokine secretion [52,53]. Heart-specific antibodies, observed in over 60% of cases of viral cardiomyopathy [40], are involved in the progression of heart disease [54]. These antibodies target structural components (such as anti-membrane, anti-sarcolemma, and anti-cytoskeleton antibodies), myosin chains (anti-myosin alpha and beta heavy chains antibodies), troponin, certain receptors (anti-acetylcholine receptor antibodies and anti-beta-1 adrenergic antibodies), mitochondrial components (NAD, creatine kinase, and pyruvate kinase), and heat shock proteins [38,54]. Viruses that damage heart tissue expose intracellular cardiac antigens and may initiate the production of heart-specific autoantibodies. Such antibodies have been identified in both experimental coxsackievirus B3 myocarditis and in patients with clinical and biopsy-confirmed myocarditis, supporting an autoimmune role in ongoing myocardial injury [51,55] (see Figure 2).

The recovery phase is the longest, lasting weeks or months [11]. This phase concludes with the restoration of cardiac function or progresses to chronic myocarditis and cardiac remodeling toward dilated cardiomyopathy [56]. The shift toward harmful remodeling is encouraged by the persistence of the virus within endothelial cells and myocytes, the inflammatory response, the balance between NK and Treg cells, and the involvement of autoimmune mechanisms [11]. Extracellular matrix remodeling is driven by pro-inflammatory cytokines such as TNF-α and IL-1β [57], which activate matrix metalloproteinases (collagenase, elastase, and gelatinase) [11]. MicroRNAs like miR-21, miR-208b, and miR-499 may also play a role in the progression of viral myocarditis to dilated cardiomyopathy [58].

COVID-19 myocarditis results from the harmful effects of the virus on cardiac myocytes and mostly the activation of immune processes, according to most authors [59,60]. In patients previously treated with glucocorticoids, the prevalence of myocarditis and myocardial inflammation was lower than in patients treated with IL-6 blockade [26]. Histology aspects are a subject of debate. In one autopsy series of 40 patients who died during the first pandemic wave in northern Italy, it was shown that neither the viral genome nor the spike protein was detected in patients with lymphocytic myocarditis, but was detected in cardiomyocytes without signs of myocarditis [61]. Some studies on hearts from deceased individuals reported no specific viral lesions despite detecting viral RNA replication [62]. Other researchers found viral RNA but not viral particles within the hearts [63] or inside cardiomyocytes [64]. Additional studies indicated that in patients who died from SARS-CoV-2 infection, the presence of SARS-CoV in the heart was linked to increased macrophage infiltration, myocardial damage, and reduced myocardial ACE2 protein levels [65].

The first step in SARS-CoV-2 myocarditis involves the virus binding to the host cell membrane, followed by its entry into the cells via endocytosis. After entry, SARS-CoV-2 releases positive-strand RNA into the cytoplasm, where it is translated into polyproteins and structural proteins. This is followed by the assembly of the viral genome and envelope. The immature virion then moves to the endoplasmic reticulum, fuses with the cell membrane, and is subsequently released from the host cell [66,67].

The primary route for viral binding is represented by angiotensin-converting enzyme 2 (ACE2). Both the binding process and viral entry processes are facilitated by the viral envelope S-protein into S1 and S2 subunits. The S1 domain binds to ACE2, while the S2 domain facilitates the fusion of the virion envelope with the cell membrane [68]. There are two forms of ACE2: a membrane-bound form, which acts as a receptor for SARS-CoV-2, and a soluble form, which neutralizes free virions by shielding the viral spike protein (S) [69]. Angiotensin II receptor type 2 (AGTR2) has a higher affinity for the spike protein than ACE2 [70].

The tropism of SARS-CoV-2 for the heart (generally for SARS-CoV viruses—Ref. [71] is explained by the fact that cardiomyocytes, pericytes, and endothelial cells strongly express the ACE2 receptor [72]. In diabetic patients and in patients with heart diseases, especially those susceptible to SARS infections, ACE2 expression is increased [73,74].

ACE2 is highly expressed by the vascular endothelium [75] and by perivascular mural cells (pericytes). Pericytes express higher levels of ACE2 than cardiomyocytes [6]. Pericytes interact with endothelial cells and neuron-like cells, which is relevant for medications that improve microcirculation and could help reduce or alleviate heart injury [6]. Regarding the expression of ACE2 in the heart, opinions vary: some authors report low levels [6,76], while others argue that ACE2 is highly expressed [77]. In any case, ACE2 levels in the heart are lower than in the intestine and kidney, but higher than in the lung [6].

Unlike smooth muscle cells, which are located in the coronary arteries or arterioles, pericytes extend outside the capillary and venules’ endothelium, suggesting that pericytes are a target for SARS-CoV-2 [6] and may contribute to endothelial cell dysfunction and microcirculatory issues.

ACE converts angiotensin I to angiotensin II and inactivates bradykinin [71]. ACE2 does not affect angiotensin II production but converts angiotensin II (Ang II) to Ang 1–7 [78], which has protective roles for the heart, as demonstrated in experimental models and humans. In ACE2 knockout mice, angiotensin II levels rise, and heart contractility decreases [79]. Downregulation of ACE2 is linked to oxidative stress, leading to impaired degradation of angiotensin II [80]. Reduced ACE2 expression is associated with age-related cardiomyopathy [65,79,81]. ACE2 levels increase in pregnant women, helping to protect them from hypertension [82].

In murine models [65], it was shown that SARS-CoV-2 reduces myocardial ACE2 mRNA expression. ACE2 downregulation was seen not only with SARS-CoV-2 but also with other viruses, such as HIV and measles [83]. The mechanisms behind the complete loss of ACE2 protein in hearts infected with SARS-CoV may involve either the activation of ADAM17/TACE by the SARS spike protein, leading to cleavage and release of ACE2 [84], or SARS-CoV binding to ACE2 in endothelial cells, which causes endocytosis of the ligand/receptor complex [85,86], resulting in intracellular degradation of ACE2. SARS-CoV-2 infection causes the loss of ACE2, decreasing protective Ang 1–7 levels and increasing angiotensin II, which then leads to cardiomyocyte autophagy and apoptosis [87,88].

Besides ACE2, SARS-CoV-2 uses other pathways to enter cells, such as zinc aminopeptidase N, CD147, and neuroplin-1. Zinc aminopeptidase N (APN) serves as a binding site for various coronaviruses [89].

CD147, a glycosylated transmembrane protein (also known as basigin or extracellular matrix metalloproteinase), is expressed in blood cells [90,91] and may function as a receptor for SARS-CoV, including SARS-CoV-2 [92,93]. The interaction between the viral spike protein and CD147 on red blood cells leads to hypoxemia and abnormal interactions with endothelial cells [94]. CD147 is highly expressed in the heart, kidney, lungs, and lymphocytes (where ACE2 expression is very low) and plays a role in lymphocytic signaling [95]. CD147 may act as a T-cell receptor for SARS-CoV-2, facilitating viral entry into T-cells [96]. CD147 expression is higher in males and in persons with obesity or chronic obstructive pulmonary disease [90], conditions associated with a worse COVID-19 prognosis. CypA is the ligand for CD147 [96]. SARS-CoV-2 promotes dysregulation of the CD147-CypA signaling pathways, which may contribute to thrombotic events, cardiac hypertrophy, and even heart failure [94].

Neuropilin-1 (NRP1) is expressed by endothelial and epithelial cells in the respiratory and olfactory systems, as well as by vagal and sensory neurons—structures that show low levels of ACE2 [97,98,99,100]. NRP1 functions as a co-receptor for vascular endothelial growth factor (VEGF) [99], playing a role in vascular permeability and angiogenesis [93]. The spike protein of SARS-CoV-2 binds to NRP1 [101]. Notably, NRP1 is highly expressed on vascular endothelial cells, where it regulates endothelial cell adhesion and permeability [96].

CD26 (DPP4) is a peptidase widely expressed in the leukocytes, lungs, and kidneys, and it plays important roles in T cell activation [102]. CD26 interacts with the S1 domain of SARS-CoV-2 [103]. These interactions are under analysis, especially in diabetic patients. CD26 dysregulation is observed in obesity and diabetes [104].

Integrins may be entry receptors for SARS-CoV-2 [96].

The cytokine storm and oxidative stress play a significant role in damage and loss of cardiomyocytes in SARS-CoV-2 infection [75]. The cytokine storm, a severe, dysregulated hyperinflammatory response, can occur in the context of various viral infections, including influenza A (H5N1/H1N1), SARS-CoV-1 and 2, MERS, viral hemorrhagic fevers, and some chronic viral diseases. It is characterized by the excessive activation of innate and adaptive immune cells, leading to systemic inflammation, endothelial activation, capillary leak, and a sepsis-like clinical picture, all associated with high mortality rates and significant morbidity [105,106,107,108,109,110].

This storm, typical of COVID-19, is marked by high levels of pro-inflammatory mediators such as IL-1β, IL-6, IL-17, and GM-CSF, all involved in the differentiation of Th17 cells [111], a subgroup of CD4+ lymphocytes [112]. IL-17 promotes the secretion of IL-6 [113]. Notably, elevated IL-6 levels are significant because they promote the differentiation of Th17 cells [113]. Until now, the functional significance of impaired Th17 response in COVID-19 remains unclear. However, tissue-resident memory-like Th17 cells (Trm17 cells) appear to play an important role in the hyperinflammation seen in severe COVID-19 cases [114]. Th17 blockers therapy may be useful for improving T cell antiviral responses against SARS-CoV-2 [115,116].

The reactive oxygen species (ROS) cause redox stress, heightened inflammation, and induce mitochondrial hyperpermeability, triggering autophagy, apoptosis, and necrosis in cardiomyocytes [75], which contribute to disease development and increased viral replication [105]. Some viral infections lead to a higher ROS production, such as SARS-CoV-2, influenza A virus, dengue virus, Zika virus, herpes simplex, hepatitis B and C viruses, human immunodeficiency virus-1, respiratory syncytial virus, and enteroviruses [105]. This effect triggers apoptosis, inflammation, and lung injury while enhancing viral replication and pathogenesis [105].

Both inflammation and oxidative stress facilitate SARS-CoV-2 entry into myocytes, cause protein oxidation, and lead to myofilament contractile dysfunction [75]. The reactive oxygen species (ROS).

The NLRP3 inflammasome contributes to SARS-CoV-2 myocarditis through IL-1b and IL-18 promotion of IL-6 release [117]. It is activated by various mechanisms, such as Ang II accumulation, ROS production, and complement cascade activation caused by SARS-CoV-2 infection, among others [114]. Suppressing the NLRP3 inflammasome may prevent cytokine storms [117], offering a potential therapeutic approach for COVID-19-related myocarditis. Examples include Anakinra—a recombinant form of the natural IL-1 receptor antagonist (IL-1Ra) that inhibits IL-1a and IL-1b activity; Canakinumab—an IL-1b-neutralizing antibody; MCC950—a specific NLRP3 inflammasome inhibitor; Colchicine—an NLRP3 inflammasome activation inhibitor; and IL-37—an inhibitor of NLRP3 inflammasome activity [118].

## 6. Vascular Involvement in COVID-19

SARS-CoV-2 causes widespread endothelitis, leading to endothelial dysfunction characterized by thrombosis, hemorrhages, and edema [62]. Evidence of endothelial injury is primarily seen in post-mortem studies of pulmonary arteries, as well as in the heart, lungs, liver, and kidneys [119,120,121]. Levels and activity of von Willebrand factor, markers of inflammation-activated endothelial dysfunction, are significantly increased [122]. Patients with severe disease exhibit a hypofibrinolytic state, indicated by elevated levels of plasminogen activator inhibitor [122].

The physiopathology of vascular involvement is multifactorial, primarily involving the direct effects of the virus on endothelial cells, the cytokine storm, the oxidative stress, the renin–angiotensin–aldosterone system (RAAS), autoimmune responses, and ferroptosis—a type of cell death caused by excessive peroxidation of polyunsaturated fatty acids due to loss of glutathione peroxidase activity [27,123,124]. The viral nucleocapsid protein stimulates infected cells to produce new pro-coagulant factors [121,125]. Oxidative stress increases the production of proinflammatory cytokines (such as TNFα, IL-6, IL-1), which cause endothelial dysfunction by decreasing endothelial nitric oxide synthase (eNOS) activity and nitric oxide (NO) production [27]) while also oxidizing NO into peroxynitrite [126], reducing NO bioavailability. The ROS and decreased NO levels impair endothelial repair and promote endothelial cell senescence [127].

SARS-CoV-2 binding to ACE2 and CD147 destroys intercellular junctions [99], leading to the exposure of subendothelial collagen and a prothrombotic state. Additionally, some ultrastructural studies have detected virus-like particles within endothelial cells [121,128], resulting in ultrastructural changes in the endothelium.

After virus entry into the endothelial cells, ACE2 is downregulated, while the ACE-Angiotensin II–AT1R pathway is amplified [71]. Angiotensin II promotes the production of pro-inflammatory cytokines (such as IL-6, TNFα, TGFβ), decreases the release and production of vasodilators (NO and prostacyclin), increases ROS generation [129], and activates macrophages, leading to the production of inflammatory cytokines [130].

Activated endothelial cells secrete monocyte chemoattractant protein 1 (MCP-1) [27]. The activated monocytes, on the one hand, induce high levels of tissue factor expression, and on the other hand, promote significant NO production (in an NOs-inducible manner), leading to thrombosis, the opening of endothelial gaps, and loss of barrier function [131]. IL-6, secreted by the endothelial cells, stimulates cytokine release, complement activation, and the expression of adhesive molecules [132,133]. IL-8 and MCP-1, secreted by endothelial cells, possess vasodilator properties, which facilitate gap junction opening [131]. Cytokines also contribute to platelet activation and leukocyte recruitment to the microcirculation [133].

## 7. Acute Myocarditis After Vaccination

With an unprecedented response, the medical world managed to produce and distribute several types of vaccines in a year since the start of the COVID pandemic. The global COVID-19 vaccination campaign has been instrumental in reducing both morbidity and mortality from SARS-CoV-2 infection. Over 10 billion doses of various COVID-19 vaccines have been administered worldwide, utilizing different platforms including mRNA technology, viral vectors, recombinant proteins, and inactivated virus preparations. Overall, the benefit–risk balance of COVID-19 vaccination remains strongly favorable at the populational level, particularly by preventing severe disease, including cardiovascular complications. However, the vaccination campaign was also associated with an increased number of reports regarding side effects. Amongst these, myocarditis and pericarditis, particularly following mRNA vaccines, have raised important clinical considerations [134,135].

Acute myocarditis is reported after vaccination against COVID-19, mainly with vaccines based on mRNA technology, with the current evidence suggesting an immune-mediated process in a small subset of predisposed individuals [136]. mRNA has the functional quality of an antigen, so that it activates the inflammatory cascade by cross-reactions between SARS-CoV-2 spike glycoproteins and myocardial proteins. The mRNA platform and lipid nanoparticle components can activate innate immune pathways and downstream inflammatory responses, in susceptible hosts [36].

The temporal association between vaccination and cardiac symptoms, coupled with characteristic imaging findings, has established a causal relationship that requires careful clinical consideration and ongoing surveillance even after the WHO declared an end to the pandemic [2,136,137,138,139,140,141]. Anyway, the mortality rate in persons with myocarditis after mRNA vaccination is lower than in those with viral infection-related myocarditis.

The incidence of vaccine-associated myocarditis varies significantly across different populations and vaccine types. An umbrella review of all systematic reviews with meta-analyses published until 2024 showed that the overall incidence ranged from 0.89 to 2.36 cases of myocarditis per 100,000 doses of vaccine administered [142]. Other authors reported a higher incidence of vaccine-associated myocarditis, as high as 3–5 cases per 100,000 vaccinated people [135,143,144], occurring mainly in the first week after vaccination, and more frequent in male subjects [36].

The meta-analysis published by Bouchlarhem et al. in 2024 [142] concluded that the overall incidence is low and does not differ from that described in the general population before vaccination [142]. Another study has shown that the pooled incidence of myocarditis/pericarditis was 4.5 (95% 3.14–6.11) per 100,000 vaccinations across all doses [145]. All in all, COVID-19 vaccination was associated with an increased risk of myocarditis or pericarditis (RR = 2.04, (5% CI = 1.33–3.14) with a higher risk in people who received the second dose. This meta-analysis included 11 studies with 58,620,611 subjects [146].

The relatively low risk of myocarditis after vaccination is in contrast to the incidence of COVID-19-associated cardiac involvement, which is estimated to be 100 times higher (1000 to 1400 cases per 100,000 people with SARS-CoV-2 infection) [147]. SARS-CoV-2 infection confers a consistently higher myocarditis risk across most ages/sexes. For example, in England, infection markedly increased myocarditis risk across age groups in the general population; while vaccine-associated risks were concentrated in young males, infection risk exceeded vaccine risk in the general population. The only exception was in men <40 years old, where a second-dose of mRNA-1273 produced more excess events per million than a positive SARS-CoV-2 test (97 [95% CI, 91–99] versus 16 [95% CI, 12–18]) [3]. Another cohort study of 23 million residents from 4 countries in the north of Europe showed that in high-risk young males, a second-dose of mRNA-1273 could yield on the order of tens per 100,000 excess events over 28 days; again, infection produced fewer excess events in that specific subgroup population but more in the general population. Among males aged 16 to 24 years, the adjusted incidence risk ratio was 12.50 for a second dose of BNT162b2 and 38.29 for a second dose of mRNA-1273 [148]. In conclusion, most studies highlight a narrow populational group for which the risk assessment and counselling are necessary.

Another important aspect when comparing infection versus vaccination is the clinical severity of the cases. In contrast to the overall mild presentation and good outcome of vaccine-associated myocarditis, COVID-19 is associated with a major risk of cardiovascular complications [147,149]. For example, a study published by Aikawa et al. [149] found that among patients with COVID-19, 10% of outpatients and 40% of hospitalized patients had clinically significant myocardial injury, while advanced age and comorbidities such as obesity, diabetes mellitus, hypertension, and renal dysfunction were the main predisposing factors [149]. Husby et al. showed that in patients aged between 12–39 y.o (who had the highest risk of developing myocarditis after vaccination), COVID-19 myocarditis was associated with an increased risk of heart failure or death within 90 days of admission (relative risk 5.78, 1.84 to 18.20) compared with myocarditis associated with vaccination [150]. Therefore, even in age groups where vaccine associated myocarditis is most discussed, vaccination continues to provide net protection by reducing infection and severe outcomes, which carry a higher and more severe cardiac risk.

Young males, especially adolescents and young adults, represent the clearest high-risk population for myocarditis following mRNA SARS-CoV-2 vaccination, as shown by multiple large population studies and active surveillance reports. The highest incidence is concentrated in males aged approximately 12–39 years, with risk peaking in the week after the second mRNA dose.

Product-specific analyses from large Nordic and national cohorts additionally showed a greater excess risk after mRNA-1273 (Moderna) than after BNT162b2 (Pfizer-BioNTech) in young males.

Another important aspect is the fact that shorter inter-dose intervals have been associated with increased myocarditis reporting.

Females of all ages had a significantly lower risk across all age groups [135,141,148]. Viral vector vaccines (ChAdOx1, Ad26.COV2.S) are associated with different risk profiles, with myocarditis being less commonly reported compared to mRNA platforms [151,152,153].

The pathophysiology of SARS-CoV-2 vaccine-associated myocarditis remains incompletely understood, and multiple mechanisms are being proposed based on current evidence. Importantly, the biological mechanisms proposed to explain vaccine-associated myocarditis remain hypotheses and should be interpreted as such.

The leading mechanistic hypotheses include:

• An aberrant innate immune activation by the vaccine platform (mRNA and lipid nanoparticle components) that triggers myocardial inflammation in predisposed individuals. The higher prevalence of myocarditis in young males may be explained by higher levels of androgens, especially testosterone, which can enhance the pro-inflammatory response by Th1 lymphocytes and pro-inflammatory cytokine production [154,155]. The lipid nanoparticles may act as adjuvants, enhancing immune responses, but could potentially contribute to excessive inflammatory reactions in susceptible individuals [156].
• An adaptive immune response in which anti-spike antibodies or spike-directed T cells cross-react with cardiac antigens. This represents the molecular mimicry hypothesis and was described by Nunez et al. [157]. The molecular mimicry is associated with a cross-reaction of antibodies against the spike protein with self-antigens such as heavy chains of myosin or troponin C1 [158].
• A hyper-inflammatory recall response after repeat antigen exposure, consistent with a short latency and robust anamnestic immunity.

However, no single mechanism has been definitively proven. Most observational studies signal that risk is higher after products containing larger mRNA payloads and that risk is concentrated in young males. This supports a model in which antigen dose, immune priming/timing, and host susceptibility (including sex-hormone-modulated immune responses) play crucial roles [136,157,159].

The management of vaccine-associated myocarditis follows established principles for acute myocarditis care, with modifications based on the typically mild and self-limiting nature of this condition [141,160,161]. Non-steroidal anti-inflammatory drugs represent the cornerstone of anti-inflammatory therapy, with patients showing clinical improvement within 24–72 h on initiation, with ibuprofen or indomethacin being commonly used. Colchicine may be considered as an alternative or adjunctive therapy, particularly in cases with concomitant pericarditis. Corticosteroids are generally reserved for severe cases or those not responding to first-line anti-inflammatory therapy. The routine use of corticosteroids is limited by concerns regarding potential interference with immune responses and unclear benefit in this specific patient population [141,154,162,163].

For patients who develop myocarditis associated with SARS-CoV-2 vaccinations, decisions regarding future doses require careful individualized risk–benefit assessment. Currently, major health organizations generally advise deferring additional doses until complete recovery, with preference for vaccines demonstrating lower myocarditis risk profiles when future vaccination is indicated. The main factors influencing future vaccination decisions include severity and duration of initial episode, complete recovery versus persistent cardiac abnormalities, individual COVID-19 risk factors and exposure risk, availability of alternative vaccine platforms, and evolving epidemiological data on breakthrough infections [141,163,164].

Future research priorities include elucidating the complete pathophysiological mechanisms, optimizing treatment protocols, establishing definitive long-term outcome data, and developing personalized risk assessment tools to guide individual vaccination decisions.

## 8. Clinical Manifestations of Cardiac Involvement

Although primarily a respiratory illness, COVID-19 is linked to various cardiac complications, both acute and long-term. The clinical presentation varies greatly depending on the extent and type of cardiac damage, preexisting risk factors and comorbidities, as well as the severity of the respiratory illness.

Acute SARS-CoV-2 infection has been linked to myocarditis, both type 1 and type 2 acute myocardial infarction, myocardial damage from multisystem inflammatory syndrome, as well as new-onset or decompensated heart failure in patients with a history of cardiovascular disease, pulmonary embolism, and arrhythmias [165,166].

In patients with COVID-19, the presence of symptoms such as disproportionate fatigue, dyspnea, palpitations, chest pain, or chest tightness during exertion should be a warning sign for systematic evaluation to exclude myocarditis. However, it should be noted that some patients experience no symptoms or deteriorate suddenly with the appearance of arrhythmias, acute heart failure, or even cardiogenic shock [1]. Most studies on COVID-19 report that myocarditis occurs more frequently in young men aged 16 to 30 years [167]. Therefore, in this patient group, specific tests are recommended in the clinical context described above.

A variety of cardiovascular symptoms, such as fatigue, shortness of breath, chest pain, and palpitations, have been reported in patients who have recovered from the acute phase of SARS-CoV-2 infection. According to the definition first established by the authors of the 2020 NICE guideline, long-term COVID refers to the persistence of symptoms more than 4 weeks after infection (NICE guideline [NG188]). This term includes two phases: the ongoing symptomatic phase (4–12 weeks) and the post-COVID-19 syndrome (>12 weeks). Pathophysiological mechanisms for long COVID are still poorly understood, but the underlying cardiovascular changes include chronic myocardial inflammation, myocardial infarction, right ventricular dysfunction, and arrhythmias [168].

Post-vaccination myocarditis is most often linked to mild symptoms that occur within the first few days after the initial or second dose of mRNA vaccine, with chest pain being the most common symptom (96% of patients) [169]. Fatigue, shortness of breath, or arrhythmias are less frequently seen in patients with vaccine-induced myocarditis. An increased risk of extrasystoles was observed, rising from 1.17 after the first dose (95% CI 1.06–1.28) to 1.22 after the second dose (1.10–1.36) [170].

## 9. Diagnosis of Cardiac Involvement

Early diagnosis and prompt treatment of cardiovascular complications related to COVID-19 are crucial for reducing morbidity and mortality caused by the disease. Therefore, in addition to a comprehensive medical history and thorough physical examination, electrocardiogram (ECG) and cardiac biomarkers (troponin I, troponin T, NT-proBNP, and BNP) serve as highly useful initial screening tools in hospitalized patients and/or those at high risk for cardiovascular events.

Studies conducted on hospitalized COVID-19 patients showed ECG changes in 53% to 85% of cases, depending on the severity of the disease [99]. The most common ECG findings were ST-T changes, followed by heart rate and rhythm abnormalities [99,171]. Besides ST-T changes, other potential ECG signs seen in patients with myocarditis include: new bundle branch block, pseudoinfarction, new-onset atrial fibrillation, QT prolongation, ventricular arrhythmia, bradyarrhythmia, advanced atrioventricular block, or ST elevation and PR depression in patients with associated pericarditis. However, these findings are not sensitive, and their absence does not rule out myocarditis [1].

Elevated cardiac troponin, caused by ischemic or non-ischemic myocardial injury, is a common finding in patients with SARS-CoV-2 infection, with a prevalence of 7% to 44% among hospitalized patients [172]. In the study by Majure et al. [173], the patients with mild or severely elevated troponin had a two to fourfold higher risk of death compared to those with normal troponin levels. The troponin level is an independent predictor of in-hospital mortality, regardless of previous cardiovascular disease, ECG changes suggestive of acute coronary syndrome, or the degree of elevation of inflammatory markers. Accordingly, the measurement of troponin level is mandatory for risk stratification and helps identify patients who may require additional imaging and closer monitoring. Notably, a normal cardiac troponin level does not rule out myocarditis, especially in cases of giant cell myocarditis or during the chronic phase of myocardial injury [172]. In one study, the sensitivity of elevated TnI for myocarditis was only 34% [174].

Natriuretic peptide (BNP and NT-proBNP) levels are elevated during COVID-19 infection as indicators of cardiac injury and dysfunction. Severe SARS-CoV-2 infection, mechanical ventilation, and hospitalization are associated with increased BNP or NT-proBNP levels, and assessing natriuretic peptide levels could help identify patients at high risk who need closer monitoring and additional cardiac imaging evaluations [175].

In patients with ECG and/or cardiac biomarker abnormalities, echocardiography is a very useful method for initial evaluation, establishing the subsequent diagnostic approach, and risk stratification. The main echocardiographic changes in patients with myocarditis may include reduced left ventricular (LV) ejection fraction, increased wall thickness due to edema, regional or diffuse wall motion abnormalities, diastolic dysfunction, right ventricular dysfunction, pericardial effusion, and reduced LV global longitudinal strain (GLS) [166]. A reduced LV ejection fraction is associated with increased inpatient mortality in patients with COVID-19 [176]. Of note, a normal LV ejection fraction does not exclude myocardial involvement. Studies have shown that cardiac involvement could be present in 52 to 70% of patients with SARS-CoV-2 infection, but the most common findings were subclinical changes, such as reduced GLS or diastolic dysfunction.

**Figure 3:**
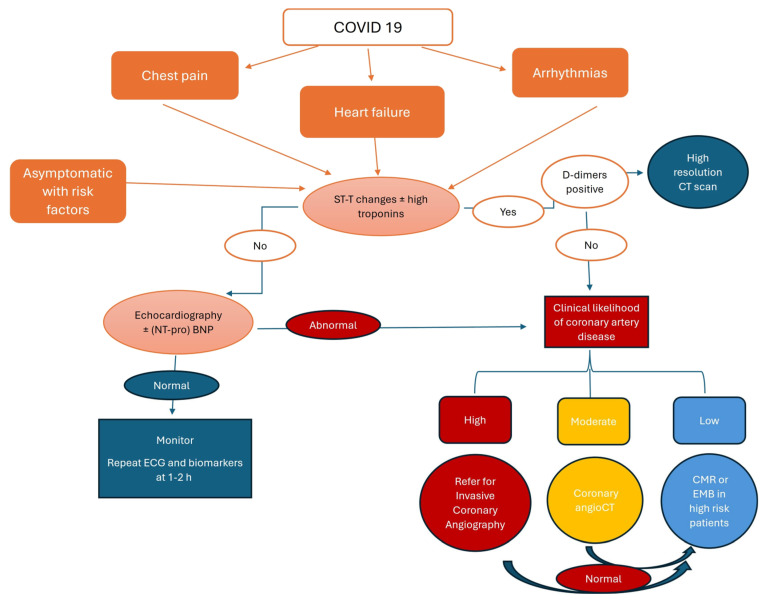
Diagnostic algorithm of cardiac involvement in COVID-19 patients based on initial clinical presentation (adapted after European Society of Cardiology guidelines for managing myocarditis and pericarditis have developed several diagnostic algorithms based on initial clinical presentation [177]). CT—computer tomography; CMR—Cardiac Magnetic Resonance; EBM—Endomyocardial biopsy. The diagnostic approach algorithm is based on initial clinical presentation as well as the presence of ECG changes in association with biomarkers (troponin and D dimers) and helps in making the differential diagnosis between decompensated heart failure, pulmonary embolism, acute coronary syndromes and myocarditis.

The latest European Society of Cardiology guidelines for managing myocarditis and pericarditis have developed several diagnostic algorithms based on initial clinical presentation [177]. We propose a diagnostic approach algorithm adapted for COVID-19 patients (see Figure 3).

The presence of acute chest pain lends itself to a differential diagnosis that should include acute coronary syndromes, myocarditis with or without associated pericarditis, or non-cardiac pain. The triad of chest pain, specific ST-T changes on ECG, and elevated troponin levels requires ruling out obstructive coronary artery disease, either through coronary angiography in patients with high clinical suspicion or by CT angiography in patients with moderate likelihood.

In patients with COVID-19 who develop new or worsening heart failure (HF), the diagnostic approach recommended by the ESC guidelines, summarized by the acronym CHAMPIT (acute Coronary syndrome, Hypertension, Mechanical causes, Arrhythmia, Pulmonary embolism, Infection, Tamponade), is very useful [177]. Acute or worsening dyspnea in patients with SARS-CoV-2 infection requires urgent computed tomography pulmonary angiography (CTPA) due to the high incidence of PE in this setting. A systematic literature review revealed an incidence of PE ranging from 0% to 1.1% in ambulatory patients, 0.9% to 8.2% in hospitalized patients, and 1.8% to 18.9% in ICU patients [178]. In patients in whom we have excluded the causes of HF have been ruled out, Cardiac Magnetic Resonance (CMR) imaging is recommended for the definitive diagnosis of infectious myocarditis. Endomyocardial biopsy (EMB) remains the gold standard for histological evaluation of suspected giant cell myocarditis in patients with rapidly progressive or refractory HF, cardiogenic shock, and/or malignant ventricular arrhythmias.

CMR is the best option for the non-invasive evaluation of stable cases and provides valuable information by characterizing changes in the magnetic properties of tissues. CMR studies in patients with COVID-19 have shown a wide range of myocarditis prevalence, from 0 to 60%, depending on the diagnostic criteria used and patient selection [179]. There are no specific changes for SARS-CoV-2 myocarditis; the diagnosis is supported based on the updated Lake Louise criteria for myocarditis [180]. Evidence of both myocardial edema (T2-based criteria: abnormal T2 mapping and T2-weighted imaging) and non-ischemic myocardial injury (T1-based criteria: abnormal native and post-contrast T1 mapping and late gadolinium enhancement) is required for the diagnosis of CMR-proven myocarditis. When only one of these two diagnostic criteria is met, the condition is classified as CMR-uncertain myocarditis, and additional signs such as pericardial involvement or global or regional left ventricular systolic dysfunction on cine imaging may help establish the diagnosis [177].

**Figure 4:**
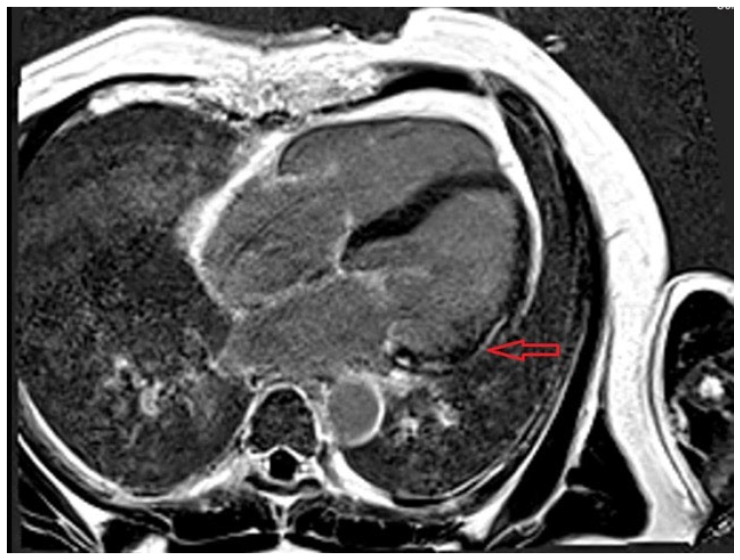
CMR-LGE 4-chamber view showing mid and basal lateral wall subepicardial scar (red arrow) in a 58-year-old patient with ventricular arrhythmia and history of COVID-19 myocarditis.

LGE burden has a poorer prognosis and predisposes to ventricular arrhythmias, heart failure, and death. Studies have shown that myocarditis with septal LGE was associated with worse outcomes [181]. In one small study, LGE was located in the basal-to-mid inferior wall in COVID-19 patients. Li et al. demonstrated that patients with COVID-19 myocarditis linked to multisystem inflammatory syndrome (MIS) exhibited more widespread myocardial inflammation and edema, unlike patients without MIS, who only showed regional lesions [182]. Additionally, the distribution of LGE was at the level of the interventricular septum in patients with MIS, while in patients without MIS, LGE was evident at the inferior ventricular wall. Twenty-five percent of patients had persistent myocardial edema at a median follow-up of 102 days, but the LGE burden decreased over time in both groups. Figure 4 displays the CMR of a patient with persistent LGE after COVID-19 myocarditis.

The same standard diagnostic approach will be used for patients with suspected vaccine-associated myocarditis. COVID-19 vaccine-associated myocarditis has a predominantly mild clinical course, with low prevalence and limited cardiac dysfunction, but myocardial injury is common, especially in adolescent males presenting after the first or second dose of an mRNA vaccine, with 82% presenting with elevated troponin levels and late gadolinium enhancement (LGE). LGE was 2.74 times more common (95% CI: 1.28, 5.83, *p* = 0.009) in older adolescents (>15 years) compared to younger patients, 3.28 times more frequent (95% CI: 0.99, 10.6, *p* = 0.052) in males versus females, 7.18 times higher (95% CI: 1.05, 49.09, *p* = 0.045) after the first dose, and 4.5 times higher (95% CI: 1.23, 16.44, *p* = 0.023) after the second dose compared to the third dose of the mRNA vaccine. LGE was observed in 60% of patients at follow-up [169].

## 10. Future Directions

Future perspectives in managing myocardial and vascular injury in patients with COVID-19 concentrate on several key areas: improved risk assessment, targeted immunotherapy, advanced diagnostic tools, and long-term monitoring. Progress in biomarker discovery and imaging is expected to enhance the early detection of cardiovascular complications and enable better risk assessment, allowing for more personalized management and improved selection of patients for vaccination, specific treatments, and close follow-up. Emerging evidence underscores the crucial role of immune dysregulation and endothelial dysfunction in COVID-19-related myocardial and vascular injury. Future treatment approaches are likely to focus on immune modulation, including agents that target specific cytokines and inflammatory pathways [183]. Long-term follow-up and preventive strategies are increasingly recognized as essential, given the risk of severe illness in some categories of patients, the cardiovascular sequelae, and post-acute COVID-19 syndromes. Research is ongoing to define optimal surveillance protocols and preventive interventions for these patients.

Finally, future management will likely incorporate lessons from ongoing and completed randomized trials, focusing on evidence-based therapeutic algorithms and multidisciplinary care models to address the complex interactions of viral, immune, and vascular factors in COVID-19-related cardiac injury [183,184].

Future efforts in managing COVID-19-related myocardial and vascular injury are also expected to benefit from advancements in multi-omics technologies, including transcriptomics, proteomics, and metabolomics. These new methods have started to identify molecular signatures linked to ongoing inflammation, endothelial dysfunction, and dysregulated immune pathways in both acute and post-acute COVID-19, providing new therapeutic targets [185,186]. The integration of omics-based biomarkers into clinical approaches may enable more accurate differentiation between immune-mediated myocardial injury, microvascular involvement, and direct viral effect on the heart. In parallel, mechanistic studies have shown potential roles for complement inhibition, antithrombotic medication, mitochondrial stabilization, and targeted endothelial protection as emerging therapeutic strategies [186,187].

## 11. Conclusions

After the COVID-19 pandemic, medical systems have been confronted with a new pathology related to complex myocardial and vascular damage, both acutely and in the long-term, both in the context of SARS-CoV-2 infection and after vaccination, especially in relation to mRNA vaccines. Although the severity and extent of these phenomena are much diminished in the post-pandemic period, understanding the intimate mechanisms behind them has important clinical implication in the diagnostic approach and the right treatment strategy, but also future perspectives for diagnosis, follow-up, treatment, and prevention.

## Data Availability

No new data were created or analyzed in this study. Data sharing does not apply to this article.

## References

[1] Siripanthong B., Nazarian S., Muser D., Deo R., Santangeli P., Khanji M.Y., Cooper L.T., Chahal C.A.A. (2020). Recognizing COVID-19-Related Myocarditis: The Possible Pathophysiology and Proposed Guideline for Diagnosis and Management. Heart Rhythm..

[2] Oster M.E., Shay D.K., Su J.R., Gee J., Creech C.B., Broder K.R., Edwards K., Soslow J.H., Dendy J.M., Schlaudecker E. (2022). Myocarditis Cases Reported After mRNA-Based COVID-19 Vaccination in the US From December 2020 to August 2021. JAMA.

[3] Patone M., Mei X.W., Handunnetthi L., Dixon S., Zaccardi F., Shankar-Hari M., Watkinson P., Khunti K., Harnden A., Coupland C.A.C. (2022). Risk of Myocarditis After Sequential Doses of COVID-19 Vaccine and SARS-CoV-2 Infection by Age and Sex. Circulation.

[4] Pellegrini D., Kawakami R., Guagliumi G., Sakamoto A., Kawai K., Gianatti A., Nasr A., Kutys R., Guo L., Cornelissen A. (2021). Microthrombi as a Major Cause of Cardiac Injury in COVID-19: A Pathologic Study. Circulation.

[5] Halushka M.K., Vander Heide R.S. (2021). Myocarditis Is Rare in COVID-19 Autopsies: Cardiovascular Findings across 277 Postmortem Examinations. Cardiovasc. Pathol..

[6] Chen L., Li X., Chen M., Feng Y., Xiong C. (2020). The ACE2 Expression in Human Heart Indicates New Potential Mechanism of Heart Injury among Patients Infected with SARS-CoV-2. Cardiovasc. Res..

[7] Huang L., Zhao P., Tang D., Zhu T., Han R., Zhan C., Liu W., Zeng H., Tao Q., Xia L. (2020). Cardiac Involvement in Patients Recovered from COVID-2019 Identified Using Magnetic Resonance Imaging. JACC Cardiovasc. Imaging.

[8] Xie Y., Xu E., Bowe B., Al-Aly Z. (2022). Long-Term Cardiovascular Outcomes of COVID-19. Nat. Med..

[9] CDC ACIP Evidence to Recommendations for Use of Moderna COVID-19 Vaccine. https://www.cdc.gov/acip/evidence-to-recommendations/bla-covid-19-moderna-etr.html.

[10] Golpour A., Patriki D., Hanson P.J., McManus B., Heidecker B. (2021). Epidemiological Impact of Myocarditis. J. Clin. Med..

[11] Kanuri S., Aedma S., Naik A., Kumar P., Mahajan P., Gupta R., Garg J., Thurugam M., Elbey A., Varadarajan P. (2023). Pathophysiology of Myocarditis: State-of-the-Art Review Corresponding Author. J. Atr. Fibrillation Electrophysiol..

[12] Fung G., Luo H., Qiu Y., Yang D., McManus B. (2016). Myocarditis. Circ. Res..

[13] Pelle M.C., Zaffina I., Lucà S., Forte V., Trapanese V., Melina M., Giofrè F., Arturi F. (2022). Endothelial Dysfunction in COVID-19: Potential Mechanisms and Possible Therapeutic Options. Life.

[14] Boehmer T.K., Kompaniyets L., Lavery A.M., Hsu J., Ko J.Y., Yusuf H., Romano S.D., Gundlapalli A.V., Oster M.E., Harris A.M. (2021). Association Between COVID-19 and Myocarditis Using Hospital-Based Administrative Data—United States, March 2020–January 2021. Morb. Mortal. Wkly. Rep..

[15] Gluckman T.J., Bhave N.M., Allen L.A., Chung E.H., Spatz E.S., Ammirati E., Baggish A.L., Bozkurt B., Cornwell W.K., Writing Committee (2022). 2022 ACC Expert Consensus Decision Pathway on Cardiovascular Sequelae of COVID-19 in Adults: Myocarditis and Other Myocardial Involvement, Post-Acute Sequelae of SARS-CoV-2 Infection, and Return to Play: A Report of the American College of Cardiology Solution Set Oversight Committee. J. Am. Coll. Cardiol..

[16] Ammirati E., Lupi L., Palazzini M., Hendren N.S., Grodin J.L., Cannistraci C.V., Schmidt M., Hekimian G., Peretto G., Bochaton T. (2022). Prevalence, Characteristics, and Outcomes of COVID-19—Associated Acute Myocarditis. Circulation.

[17] Fu L., Liu X., Su Y., Ma J., Hong K. (2020). Prevalence and Impact of Cardiac Injury on COVID-19: A Systematic Review and Meta-analysis. Clin. Cardiol..

[18] Zuin M., Rigatelli G., Battisti V., Costola G., Roncon L., Bilato C. (2023). Increased Risk of Acute Myocardial Infarction after COVID-19 Recovery: A Systematic Review and Meta-Analysis. Int. J. Cardiol..

[19] Katsoularis I., Fonseca-Rodríguez O., Farrington P., Lindmark K., Fors Connolly A.-M. (2021). Risk of Acute Myocardial Infarction and Ischaemic Stroke Following COVID-19 in Sweden: A Self-Controlled Case Series and Matched Cohort Study. Lancet.

[20] Woodruff R.C., Garg S., George M.G., Patel K., Jackson S.L., Loustalot F., Wortham J.M., Taylor C.A., Whitaker M., Reingold A. (2023). Acute Cardiac Events During COVID-19-Associated Hospitalizations. J. Am. Coll. Cardiol..

[21] Basso C., Leone O., Rizzo S., De Gaspari M., van der Wal A.C., Aubry M.-C., Bois M.C., Lin P.T., Maleszewski J.J., Stone J.R. (2020). Pathological Features of COVID-19-Associated Myocardial Injury: A Multicentre Cardiovascular Pathology Study. Eur. Heart J..

[22] Liu J., Deswal A., Khalid U. (2021). COVID-19 Myocarditis and Long-Term Heart Failure Sequelae. Curr. Opin. Cardiol..

[23] Carsana L., Sonzogni A., Nasr A., Rossi R.S., Pellegrinelli A., Zerbi P., Rech R., Colombo R., Antinori S., Corbellino M. (2020). Pulmonary Post-Mortem Findings in a Series of COVID-19 Cases from Northern Italy: A Two-Centre Descriptive Study. Lancet Infect. Dis..

[24] Zhou B., Zhao W., Feng R., Zhang X., Li X., Zhou Y., Peng L., Li Y., Zhang J., Luo J. (2020). The Pathological Autopsy of Coronavirus Disease 2019 (COVID-2019) in China: A Review. Pathog. Dis..

[25] Schaller T., Hirschbühl K., Burkhardt K., Braun G., Trepel M., Märkl B., Claus R. (2020). Postmortem Examination of Patients with COVID-19. JAMA.

[26] Bearse M., Hung Y.P., Krauson A.J., Bonanno L., Boyraz B., Harris C.K., Helland T.L., Hilburn C.F., Hutchison B., Jobbagy S. (2021). Factors Associated with Myocardial SARS-CoV-2 Infection, Myocarditis, and Cardiac Inflammation in Patients with COVID-19. Mod. Pathol..

[27] Otifi H.M., Adiga B.K. (2022). Endothelial Dysfunction in COVID-19 Infection. Am. J. Med. Sci..

[28] Fox S.E., Akmatbekov A., Harbert J.L., Li G., Quincy Brown J., Vander Heide R.S. (2020). Pulmonary and Cardiac Pathology in African American Patients with COVID-19: An Autopsy Series from New Orleans. Lancet Respir. Med..

[29] Yao X.H., Li T.Y., He Z.C., Ping Y.F., Liu H.W., Yu S.C., Mou H.M., Wang L.H., Zhang H.R., Fu W.J. (2020). A pathological report of three COVID-19 cases by minimal invasive autopsies. Zhonghua Bing Li Xue Za Zhi.

[30] Lindner D., Fitzek A., Bräuninger H., Aleshcheva G., Edler C., Meissner K., Scherschel K., Kirchhof P., Escher F., Schultheiss H.-P. (2020). Association of Cardiac Infection with SARS-CoV-2 in Confirmed COVID-19 Autopsy Cases. JAMA Cardiol..

[31] Yu S., Xu J., Yu C., Zhang X., Cheng Y., Lin D., Yan C., Guo M., Li J., He P. (2024). Persistence of SARS-CoV-2 Colonization and High Expression of Inflammatory Factors in Cardiac Tissue 6 Months after COVID-19 Recovery: A Prospective Cohort Study. Cardiovasc. Diagn. Ther..

[32] Alfieri L., Franceschetti L., Frisoni P., Bonato O., Radaelli D., Bonuccelli D., D’Errico S., Neri M. (2024). Cardiac SARS-CoV-2 Infection, Involvement of Cytokines in Postmortem Immunohistochemical Study. Diagnostics.

[33] Yu B., Wu Y., Song X., Liu G., Wang F., Zhang F., Liang B. (2023). Possible Mechanisms of SARS-CoV2-Mediated Myocardial Injury. Cardiovasc. Innov. Appl..

[34] Huber S.A., Gauntt C.J., Sakkinen P. (1998). Enteroviruses and Myocarditis: Viral Pathogenesis through Replication, Cytokine Induction, and Immunopathogenicity. Adv. Virus Res..

[35] Malkiel S., Kuan A.P., Diamond B. (1996). Autoimmunity in Heart Disease: Mechanisms and Genetic Susceptibility. Mol. Med. Today.

[36] Sozzi F.B., Gherbesi E., Faggiano A., Gnan E., Maruccio A., Schiavone M., Iacuzio L., Carugo S. (2022). Viral Myocarditis: Classification, Diagnosis, and Clinical Implications. Front. Cardiovasc. Med..

[37] Pollack A., Kontorovich A.R., Fuster V., Dec G.W. (2015). Viral Myocarditis—Diagnosis, Treatment Options, and Current Controversies. Nat. Rev. Cardiol..

[38] Maisch B. (2019). Cardio-Immunology of Myocarditis: Focus on Immune Mechanisms and Treatment Options. Front. Cardiovasc. Med..

[39] Heymans S., Eriksson U., Lehtonen J., Cooper L.T. (2016). The Quest for New Approaches in Myocarditis and Inflammatory Cardiomyopathy. J. Am. Coll. Cardiol..

[40] Tschöpe C., Ammirati E., Bozkurt B., Caforio A.L.P., Cooper L.T., Felix S.B., Hare J.M., Heidecker B., Heymans S., Hübner N. (2021). Myocarditis and Inflammatory Cardiomyopathy: Current Evidence and Future Directions. Nat. Rev. Cardiol..

[41] Leuschner F., Rauch P.J., Ueno T., Gorbatov R., Marinelli B., Lee W.W., Dutta P., Wei Y., Robbins C., Iwamoto Y. (2012). Rapid Monocyte Kinetics in Acute Myocardial Infarction Are Sustained by Extramedullary Monocytopoiesis. J. Exp. Med..

[42] Bruestle K., Hackner K., Kreye G., Heidecker B. (2020). Autoimmunity in Acute Myocarditis: How Immunopathogenesis Steers New Directions for Diagnosis and Treatment. Curr. Cardiol. Rep..

[43] Higuchi H., Hara M., Yamamoto K., Miyamoto T., Kinoshita M., Yamada T., Uchiyama K., Matsumori A. (2008). Mast Cells Play a Critical Role in the Pathogenesis of Viral Myocarditis. Circulation.

[44] Weckbach L.T., Grabmaier U., Uhl A., Gess S., Boehm F., Zehrer A., Pick R., Salvermoser M., Czermak T., Pircher J. (2019). Midkine Drives Cardiac Inflammation by Promoting Neutrophil Trafficking and NETosis in Myocarditis. J. Exp. Med..

[45] Rivadeneyra L., Charó N., Kviatcovsky D., de la Barrera S., Gómez R.M., Schattner M. (2018). Role of Neutrophils in CVB3 Infection and Viral Myocarditis. J. Mol. Cell. Cardiol..

[46] Grabie N., Hsieh D.T., Buono C., Westrich J.R., Allen J.A., Pang H., Stavrakis G., Lichtman A.H. (2003). Neutrophils Sustain Pathogenic CD8^+^ T Cell Responses in the Heart. Am. J. Pathol..

[47] Ong S., Rose N.R., Čiháková D. (2017). Natural Killer Cells in Inflammatory Heart Disease. Clin. Immunol..

[48] Liu P.P., Mason J.W. (2001). Advances in the Understanding of Myocarditis. Circulation.

[49] De Luca G., Cavalli G., Campochiaro C., Tresoldi M., Dagna L. (2018). Myocarditis: An Interleukin-1-Mediated Disease?. Front. Immunol..

[50] Vdovenko D., Eriksson U. (2018). Regulatory Role of CD4^+^ T Cells in Myocarditis. J. Immunol. Res..

[51] Myers J.M., Cooper L.T., Kem D.C., Stavrakis S., Kosanke S.D., Shevach E.M., Fairweather D., Stoner J.A., Cox C.J., Cunningham M.W. (2016). Cardiac Myosin-Th17 Responses Promote Heart Failure in Human Myocarditis. JCI Insight.

[52] Rose N.R. (2011). Critical Cytokine Pathways to Cardiac Inflammation. J. Interferon Cytokine Res..

[53] Matsumori A. (1996). Cytokines in Myocarditis and Cardiomyopathies. Curr. Opin. Cardiol..

[54] Mason J.W. (2003). Myocarditis and Dilated Cardiomyopathy: An Inflammatory Link. Cardiovasc. Res..

[55] Ying C. (2024). Viral Myocarditis. Yale J. Biol. Med..

[56] Baldeviano G.C., Barin J.G., Talor M.V., Srinivasan S., Bedja D., Zheng D., Gabrielson K., Iwakura Y., Rose N.R., Cihakova D. (2010). Interleukin-17A Is Dispensable for Myocarditis but Essential for the Progression to Dilated Cardiomyopathy. Circ. Res..

[57] Elamm C., Fairweather D., Cooper L.T. (2012). Republished: Pathogenesis and Diagnosis of Myocarditis. Postgrad. Med. J..

[58] van den Hoogen P., van den Akker F., Deddens J.C., Sluijter J.P.G. (2015). Heart Failure in Chronic Myocarditis: A Role for microRNAs?. Curr. Genom..

[59] Knowlton K.U. (2020). Pathogenesis of SARS-CoV-2 Induced Cardiac Injury from the Perspective of the Virus. J. Mol. Cell. Cardiol..

[60] Mansueto G., Niola M., Napoli C. (2020). Can COVID 2019 Induce a Specific Cardiovascular Damage or It Exacerbates Pre-Existing Cardiovascular Diseases?. Pathol. Res. Pract..

[61] Dal Ferro M., Bussani R., Paldino A., Nuzzi V., Collesi C., Zentilin L., Schneider E., Correa R., Silvestri F., Zacchigna S. (2021). SARS-CoV-2, Myocardial Injury and Inflammation: Insights from a Large Clinical and Autopsy Study. Clin. Res. Cardiol..

[62] Trypsteen W., Cleemput J.V., van Snippenberg W., Gerlo S., Vandekerckhove L. (2020). On the Whereabouts of SARS-CoV-2 in the Human Body: A Systematic Review. PLoS Pathog..

[63] Bradley B.T., Maioli H., Johnston R., Chaudhry I., Fink S.L., Xu H., Najafian B., Deutsch G., Lacy J.M., Williams T. (2020). Histopathology and Ultrastructural Findings of Fatal COVID-19 Infections in Washington State: A Case Series. Lancet.

[64] Tavazzi G., Pellegrini C., Maurelli M., Belliato M., Sciutti F., Bottazzi A., Sepe P.A., Resasco T., Camporotondo R., Bruno R. (2020). Myocardial Localization of Coronavirus in COVID-19 Cardiogenic Shock. Eur. J. Heart Fail..

[65] Oudit G.Y., Kassiri Z., Jiang C., Liu P.P., Poutanen S.M., Penninger J.M., Butany J. (2009). SARS-Coronavirus Modulation of Myocardial ACE2 Expression and Inflammation in Patients with SARS. Eur. J. Clin. Investig..

[66] Jackson C.B., Farzan M., Chen B., Choe H. (2022). Mechanisms of SARS-CoV-2 Entry into Cells. Nat. Rev. Mol. Cell Biol..

[67] Subong B.J.J., Forteza I.L. (2025). SARS-CoV-2 Replication Revisited: Molecular Insights and Current and Emerging Antiviral Strategies. COVID.

[68] Walls A.C., Park Y.-J., Tortorici M.A., Wall A., McGuire A.T., Veesler D. (2020). Structure, Function, and Antigenicity of the SARS-CoV-2 Spike Glycoprotein. Cell.

[69] Ciaglia E., Vecchione C., Puca A.A. (2020). COVID-19 Infection and Circulating ACE2 Levels: Protective Role in Women and Children. Front. Pediatr..

[70] Cui C., Huang C., Zhou W., Ji X., Zhang F., Wang L., Zhou Y., Cui Q. (2021). AGTR2, One Possible Novel Key Gene for the Entry of SARS-CoV-2 Into Human Cells. IEEE/ACM Trans. Comput. Biol. Bioinform..

[71] Turner A.J., Hiscox J.A., Hooper N.M. (2004). ACE2: From Vasopeptidase to SARS Virus Receptor. Trends Pharmacol. Sci..

[72] Topol E.J. (2020). COVID-19 Can Affect the Heart. Science.

[73] Wong D.W., Oudit G.Y., Reich H., Kassiri Z., Zhou J., Liu Q.C., Backx P.H., Penninger J.M., Herzenberg A.M., Scholey J.W. (2007). Loss of Angiotensin-Converting Enzyme-2 (Ace2) Accelerates Diabetic Kidney Injury. Am. J. Pathol..

[74] Nicin L., Abplanalp W.T., Mellentin H., Kattih B., Tombor L., John D., Schmitto J.D., Heineke J., Emrich F., Arsalan M. (2020). Cell Type-Specific Expression of the Putative SARS-CoV-2 Receptor ACE2 in Human Hearts. Eur. Heart J..

[75] Tangos M., Budde H., Kolijn D., Sieme M., Zhazykbayeva S., Lódi M., Herwig M., Gömöri K., Hassoun R., Robinson E.L. (2022). SARS-CoV-2 Infects Human Cardiomyocytes Promoted by Inflammation and Oxidative Stress. Int. J. Cardiol..

[76] Sungnak W., Huang N., Bécavin C., Berg M., Queen R., Litvinukova M., Talavera-López C., Maatz H., Reichart D., Sampaziotis F. (2020). SARS-CoV-2 Entry Factors Are Highly Expressed in Nasal Epithelial Cells Together with Innate Immune Genes. Nat. Med..

[77] Lu D., Chatterjee S., Xiao K., Riedel I., Wang Y., Foo R., Bär C., Thum T. (2020). MicroRNAs Targeting the SARS-CoV-2 Entry Receptor ACE2 in Cardiomyocytes. J. Mol. Cell. Cardiol..

[78] Scialo F., Daniele A., Amato F., Pastore L., Matera M.G., Cazzola M., Castaldo G., Bianco A. (2020). ACE2: The Major Cell Entry Receptor for SARS-CoV-2. Lung.

[79] Crackower M.A., Sarao R., Oudit G.Y., Yagil C., Kozieradzki I., Scanga S.E., Oliveira-dos-Santos A.J., da Costa J., Zhang L., Pei Y. (2002). Angiotensin-Converting Enzyme 2 Is an Essential Regulator of Heart Function. Nature.

[80] Khan Z., Shen X.Z., Bernstein E.A., Giani J.F., Eriguchi M., Zhao T.V., Gonzalez-Villalobos R.A., Fuchs S., Liu G.Y., Bernstein K.E. (2017). Angiotensin-Converting Enzyme Enhances the Oxidative Response and Bactericidal Activity of Neutrophils. Blood.

[81] Oudit G.Y., Kassiri Z., Patel M.P., Chappell M., Butany J., Backx P.H., Tsushima R.G., Scholey J.W., Khokha R., Penninger J.M. (2007). Angiotensin II-Mediated Oxidative Stress and Inflammation Mediate the Age-Dependent Cardiomyopathy in ACE2 Null Mice. Cardiovasc. Res..

[82] Brosnihan K.B., Neves L.A.A., Joyner J., Averill D.B., Chappell M.C., Sarao R., Penninger J., Ferrario C.M. (2003). Enhanced Renal Immunocytochemical Expression of ANG-(1-7) and ACE2 during Pregnancy. Hypertension.

[83] Kuba K., Imai Y., Rao S., Gao H., Guo F., Guan B., Huan Y., Yang P., Zhang Y., Deng W. (2005). A Crucial Role of Angiotensin Converting Enzyme 2 (ACE2) in SARS Coronavirus-Induced Lung Injury. Nat. Med..

[84] Haga S., Yamamoto N., Nakai-Murakami C., Osawa Y., Tokunaga K., Sata T., Yamamoto N., Sasazuki T., Ishizaka Y. (2008). Modulation of TNF-α-Converting Enzyme by the Spike Protein of SARS-CoV and ACE2 Induces TNF-α Production and Facilitates Viral Entry. Proc. Natl. Acad. Sci. USA.

[85] Peiris J.S.M., Guan Y., Yuen K.Y. (2004). Severe Acute Respiratory Syndrome. Nat. Med..

[86] Wang S., Guo F., Liu K., Wang H., Rao S., Yang P., Jiang C. (2008). Endocytosis of the Receptor-Binding Domain of SARS-CoV Spike Protein Together with Virus Receptor ACE2. Virus Res..

[87] Lin L., Liu X., Xu J., Weng L., Ren J., Ge J., Zou Y. (2016). Mas Receptor Mediates Cardioprotection of Angiotensin-(1-7) against Angiotensin II-Induced Cardiomyocyte Autophagy and Cardiac Remodelling through Inhibition of Oxidative Stress. J. Cell. Mol. Med..

[88] Huang C.-Y., Kuo W.-W., Yeh Y.-L., Ho T.-J., Lin J.-Y., Lin D.-Y., Chu C.-H., Tsai F.-J., Tsai C.-H., Huang C.-Y. (2014). ANG II Promotes IGF-IIR Expression and Cardiomyocyte Apoptosis by Inhibiting HSF1 via JNK Activation and SIRT1 Degradation. Cell Death Differ..

[89] Yeager C.L., Ashmun R.A., Williams R.K., Cardellichio C.B., Shapiro L.H., Look A.T., Holmes K.V. (1992). Human Aminopeptidase N Is a Receptor for Human Coronavirus 229E. Nature.

[90] Faghihi H. (2020). CD147 as an Alternative Binding Site for the Spike Protein on the Surface of SARS-CoV-2. Eur. Rev. Med. Pharmacol. Sci..

[91] Shilts J., Crozier T.W.M., Greenwood E.J.D., Lehner P.J., Wright G.J. (2021). No Evidence for Basigin/CD147 as a Direct SARS-CoV-2 Spike Binding Receptor. Sci. Rep..

[92] Guo H.-F., Vander Kooi C.W. (2015). Neuropilin Functions as an Essential Cell Surface Receptor. J. Biol. Chem..

[93] Roy S., Bag A.K., Singh R.K., Talmadge J.E., Batra S.K., Datta K. (2017). Multifaceted Role of Neuropilins in the Immune System: Potential Targets for Immunotherapy. Front. Immunol..

[94] Loh D. (2020). The Potential of Melatonin in the Prevention and Attenuation of Oxidative Hemolysis and Myocardial Injury from Cd147 SARS-CoV-2 Spike Protein Receptor Binding. Melatonin Res..

[95] Yurchenko V., Constant S., Eisenmesser E., Bukrinsky M. (2010). Cyclophilin–CD147 Interactions: A New Target for Anti-Inflammatory Therapeutics. Clin. Exp. Immunol..

[96] Alipoor S.D., Mirsaeidi M. (2022). SARS-CoV-2 Cell Entry beyond the ACE2 Receptor. Mol. Biol. Rep..

[97] Cantuti-Castelvetri L., Ojha R., Pedro L.D., Djannatian M., Franz J., Kuivanen S., van der Meer F., Kallio K., Kaya T., Anastasina M. (2020). Neuropilin-1 Facilitates SARS-CoV-2 Cell Entry and Infectivity. Science.

[98] Mayi B.S., Leibowitz J.A., Woods A.T., Ammon K.A., Liu A.E., Raja A. (2021). The Role of Neuropilin-1 in COVID-19. PLoS Pathog..

[99] Wang Y., Chen L., Wang J., He X., Huang F., Chen J., Yang X. (2020). Electrocardiogram Analysis of Patients with Different Types of COVID-19. Ann. Noninvasive Electrocardiol..

[100] Davies J., Randeva H.S., Chatha K., Hall M., Spandidos D.A., Karteris E., Kyrou I. (2020). Neuropilin-1 as a New Potential SARS-CoV-2 Infection Mediator Implicated in the Neurologic Features and Central Nervous System Involvement of COVID-19. Mol. Med. Rep..

[101] Daly J.L., Simonetti B., Klein K., Chen K.-E., Williamson M.K., Antón-Plágaro C., Shoemark D.K., Simón-Gracia L., Bauer M., Hollandi R. (2020). Neuropilin-1 Is a Host Factor for SARS-CoV-2 Infection. Science.

[102] Fleischer B. (1994). CD26: A Surface Protease Involved in T-Cell Activation. Immunol. Today.

[103] Vankadari N., Wilce J.A. (2020). Emerging COVID-19 Coronavirus: Glycan Shield and Structure Prediction of Spike Glycoprotein and Its Interaction with Human CD26. Emerg. Microbes Infect..

[104] Röhrborn D., Wronkowitz N., Eckel J. (2015). DPP4 in Diabetes. Front. Immunol..

[105] Kayesh M.E.H., Kohara M., Tsukiyama-Kohara K. (2025). Effects of Oxidative Stress on Viral Infections: An Overview. npj Viruses.

[106] Lu Q., Ding Y., Liu W., Liu S. (2025). Viral Infections and the Glutathione Peroxidase Family: Mechanisms of Disease Development. Antioxid. Redox Signal..

[107] Tang X.-D., Ji T.-T., Dong J.-R., Feng H., Chen F.-Q., Chen X., Zhao H.-Y., Chen D.-K., Ma W.-T. (2021). Pathogenesis and Treatment of Cytokine Storm Induced by Infectious Diseases. Int. J. Mol. Sci..

[108] Kombe Kombe A.J., Fotoohabadi L., Gerasimova Y., Nanduri R., Lama Tamang P., Kandala M., Kelesidis T. (2024). The Role of Inflammation in the Pathogenesis of Viral Respiratory Infections. Microorganisms.

[109] Nie J., Zhou L., Tian W., Liu X., Yang L., Yang X., Zhang Y., Wei S., Wang D.W., Wei J. (2025). Deep Insight into Cytokine Storm: From Pathogenesis to Treatment. Signal Transduct. Target. Ther..

[110] Ragab D., Salah Eldin H., Taeimah M., Khattab R., Salem R. (2020). The COVID-19 Cytokine Storm; What We Know So Far. Front. Immunol..

[111] Xu Z., Shi L., Wang Y., Zhang J., Huang L., Zhang C., Liu S., Zhao P., Liu H., Zhu L. (2020). Pathological Findings of COVID-19 Associated with Acute Respiratory Distress Syndrome. Lancet Respir. Med..

[112] Paiva I.A., Badolato-Corrêa J., Familiar-Macedo D., de-Oliveira-Pinto L.M. (2021). Th17 Cells in Viral Infections—Friend or Foe?. Cells.

[113] Coomes E.A., Haghbayan H. (2020). Interleukin-6 in COVID-19: A Systematic Review and Meta-Analysis. Rev. Med. Virol..

[114] Zhao Y., Kilian C., Turner J.-E., Bosurgi L., Roedl K., Bartsch P., Gnirck A.-C., Cortesi F., Schultheiß C., Hellmig M. (2021). Clonal Expansion and Activation of Tissue-Resident Memory-like T_H_17 Cells Expressing GM-CSF in the Lungs of Patients with Severe COVID-19. Sci. Immunol..

[115] Zeisel M.B., Felmlee D.J., Baumert T.F. (2013). Hepatitis C Virus Entry. Curr. Top. Microbiol. Immunol..

[116] Helenius A. (2018). Virus Entry: Looking Back and Moving Forward. J. Mol. Biol..

[117] Ratajczak M.Z., Bujko K., Ciechanowicz A., Sielatycka K., Cymer M., Marlicz W., Kucia M. (2021). SARS-CoV-2 Entry Receptor ACE2 Is Expressed on Very Small CD45^−^ Precursors of Hematopoietic and Endothelial Cells and in Response to Virus Spike Protein Activates the Nlrp3 Inflammasome. Stem Cell Rev. Rep..

[118] Xu J., Zhou Z., Zheng Y., Yang S., Huang K., Li H. (2023). Roles of Inflammasomes in Viral Myocarditis. Front. Cell. Infect. Microbiol..

[119] Copin M.-C., Parmentier E., Duburcq T., Poissy J., Mathieu D., Lille COVID-19 ICU and Anatomopathology Group (2020). Time to Consider Histologic Pattern of Lung Injury to Treat Critically Ill Patients with COVID-19 Infection. Intensive Care Med..

[120] Su H., Yang M., Wan C., Yi L.-X., Tang F., Zhu H.-Y., Yi F., Yang H.-C., Fogo A.B., Nie X. (2020). Renal Histopathological Analysis of 26 Postmortem Findings of Patients with COVID-19 in China. Kidney Int..

[121] Varga Z., Flammer A.J., Steiger P., Haberecker M., Andermatt R., Zinkernagel A.S., Mehra M.R., Schuepbach R.A., Ruschitzka F., Moch H. (2020). Endothelial Cell Infection and Endotheliitis in COVID-19. Lancet.

[122] Pons S., Fodil S., Azoulay E., Zafrani L. (2020). The Vascular Endothelium: The Cornerstone of Organ Dysfunction in Severe SARS-CoV-2 Infection. Crit. Care.

[123] Jacobs W., Lammens M., Kerckhofs A., Voets E., Van San E., Van Coillie S., Peleman C., Mergeay M., Sirimsi S., Matheeussen V. (2020). Fatal Lymphocytic Cardiac Damage in Coronavirus Disease 2019 (COVID-19): Autopsy Reveals a Ferroptosis Signature. ESC Heart Fail..

[124] Loo J., Spittle D.A., Newnham M. (2021). COVID-19, Immunothrombosis and Venous Thromboembolism: Biological Mechanisms. Thorax.

[125] Keller T.T., Mairuhu A.T.A., de Kruif M.D., Klein S.K., Gerdes V.E.A., ten Cate H., Brandjes D.P.M., Levi M., van Gorp E.C.M. (2003). Infections and Endothelial Cells. Cardiovasc. Res..

[126] Esper R.J., Nordaby R.A., Vilariño J.O., Paragano A., Cacharrón J.L., Machado R.A. (2006). Endothelial Dysfunction: A Comprehensive Appraisal. Cardiovasc. Diabetol..

[127] Loomans C.J.M., Wan H., de Crom R., van Haperen R., de Boer H.C., Leenen P.J.M., Drexhage H.A., Rabelink T.J., van Zonneveld A.J., Staal F.J.T. (2006). Angiogenic Murine Endothelial Progenitor Cells Are Derived from a Myeloid Bone Marrow Fraction and Can Be Identified by Endothelial NO Synthase Expression. Arterioscler. Thromb. Vasc. Biol..

[128] Ackermann M., Verleden S.E., Kuehnel M., Haverich A., Welte T., Laenger F., Vanstapel A., Werlein C., Stark H., Tzankov A. (2020). Pulmonary Vascular Endothelialitis, Thrombosis, and Angiogenesis in Covid-19. N. Engl. J. Med..

[129] Chakrabarti S., Wu J. (2016). Bioactive Peptides on Endothelial Function. Food Sci. Hum. Wellness.

[130] Verdecchia P., Cavallini C., Spanevello A., Angeli F. (2020). The Pivotal Link between ACE2 Deficiency and SARS-CoV-2 Infection. Eur. J. Intern. Med..

[131] Opal S.M., van der Poll T. (2015). Endothelial Barrier Dysfunction in Septic Shock. J. Intern. Med..

[132] Desai T.R., Leeper N.J., Hynes K.L., Gewertz B.L. (2002). Interleukin-6 Causes Endothelial Barrier Dysfunction via the Protein Kinase C Pathway. J. Surg. Res..

[133] Hunter C.A., Jones S.A. (2015). IL-6 as a Keystone Cytokine in Health and Disease. Nat. Immunol..

[134] Husby A., Madsen M., Andersen M., Torp-Pedersen C. (2021). SARS-CoV-2 Vaccination and Myocarditis or Myopericarditis: Population Based Cohort Study. BMJ.

[135] Mevorach D., Anis E., Cedar N., Bromberg M., Haas E.J., Nadir E., Olsha-Castell S., Arad D., Hasin T., Levi N. (2021). Myocarditis after BNT162b2 mRNA Vaccine against Covid-19 in Israel. N. Engl. J. Med..

[136] Bozkurt B., Kamat I., Hotez P.J. (2021). Myocarditis with COVID-19 mRNA Vaccines. Circulation.

[137] Patone M., Mei X.W., Handunnetthi L., Dixon S., Zaccardi F., Shankar-Hari M., Watkinson P., Khunti K., Harnden A., Coupland C.A.C. (2022). Risks of Myocarditis, Pericarditis, and Cardiac Arrhythmias Associated with COVID-19 Vaccination or SARS-CoV-2 Infection. Nat. Med..

[138] Gargano J.W., Wallace M., Hadler S.C., Langley G., Su J.R., Oster M.E., Broder K.R., Gee J., Weintraub E., Shimabukuro T. (2021). Use of mRNA COVID-19 Vaccine After Reports of Myocarditis Among Vaccine Recipients: Update from the Advisory Committee on Immunization Practices—United States, June 2021. Morb. Mortal. Wkly. Rep..

[139] Diaz G.A., Parsons G.T., Gering S.K., Meier A.R., Hutchinson I.V., Robicsek A. (2021). Myocarditis and Pericarditis After Vaccination for COVID-19. JAMA.

[140] Verma A.K., Lavine K.J., Lin C.-Y. (2021). Myocarditis after Covid-19 mRNA Vaccination. N. Engl. J. Med..

[141] CDC Clinical Considerations: Myocarditis After COVID-19 Vaccines. https://www.cdc.gov/vaccines/covid-19/clinical-considerations/myocarditis.html.

[142] Bouchlarhem A., Boulouiz S., Bazid Z., Ismaili N., El Ouafi N. (2024). Is There a Causal Link Between Acute Myocarditis and COVID-19 Vaccination: An Umbrella Review of Published Systematic Reviews and Meta-Analyses. Clin. Med. Insights Cardiol..

[143] Witberg G., Barda N., Hoss S., Richter I., Wiessman M., Aviv Y., Grinberg T., Auster O., Dagan N., Balicer R.D. (2021). Myocarditis after COVID-19 Vaccination in a Large Health Care Organization. N. Engl. J. Med..

[144] Mahasing C., Doungngern P., Jaipong R., Nonmuti P., Chimmanee J., Wongsawat J., Boonyasirinant T., Wanlapakorn C., Leelapatana P., Yingchoncharoen T. (2023). Myocarditis and Pericarditis Following COVID-19 Vaccination in Thailand. Vaccines.

[145] Katoto P.D.M.C., Byamungu L.N., Brand A.S., Tamuzi J.L., Kakubu M.A.M., Wiysonge C.S., Gray G. (2023). Systematic Review and Meta-Analysis of Myocarditis and Pericarditis in Adolescents Following COVID-19 BNT162b2 Vaccination. NPJ Vaccines.

[146] Gao J., Feng L., Li Y., Lowe S., Guo Z., Bentley R., Xie C., Wu B., Xie P., Xia W. (2023). A Systematic Review and Meta-Analysis of the Association Between SARS-CoV-2 Vaccination and Myocarditis or Pericarditis. Am. J. Prev. Med..

[147] Maiese A., Frati P., Del Duca F., Santoro P., Manetti A.C., La Russa R., Di Paolo M., Turillazzi E., Fineschi V. (2021). Myocardial Pathology in COVID-19-Associated Cardiac Injury: A Systematic Review. Diagnostics.

[148] Karlstad Ø., Hovi P., Husby A., Härkänen T., Selmer R.M., Pihlström N., Hansen J.V., Nohynek H., Gunnes N., Sundström A. (2022). SARS-CoV-2 Vaccination and Myocarditis in a Nordic Cohort Study of 23 Million Residents. JAMA Cardiol..

[149] Aikawa T., Takagi H., Ishikawa K., Kuno T. (2021). Myocardial Injury Characterized by Elevated Cardiac Troponin and In-Hospital Mortality of COVID-19: An Insight from a Meta-Analysis. J. Med. Virol..

[150] Husby A., Gulseth H.L., Hovi P., Hansen J.V., Pihlström N., Gunnes N., Härkänen T., Dahl J., Karlstad Ø., Heliö T. (2023). Clinical Outcomes of Myocarditis after SARS-CoV-2 mRNA Vaccination in Four Nordic Countries: Population Based Cohort Study. BMJ Med..

[151] Chouchana L., Blet A., Al-Khalaf M., Kafil T.S., Nair G., Robblee J., Drici M., Valnet-Rabier M., Micallef J., Salvo F. (2022). Features of Inflammatory Heart Reactions Following mRNA COVID-19 Vaccination at a Global Level. Clin. Pharmacol. Ther..

[152] Choi M.J., Na Y., Hyun H.J., Nham E., Yoon J.G., Seong H., Seo Y.B., Choi W.S., Song J.Y., Kim D.W. (2024). Comparative Safety Analysis of mRNA and Adenoviral Vector COVID-19 Vaccines: A Nationwide Cohort Study Using an Emulated Target Trial Approach. Clin. Microbiol. Infect..

[153] Ha J., Song M.C., Park S., Kang H., Kyung T., Kim N., Kim D.K., Bae K., Lee K.J., Lee E. (2024). Deciphering Deaths Associated with Severe Serious Adverse Events Following SARS-CoV-2 Vaccination: A Retrospective Cohort Study. Vaccine X.

[154] Florek K., Sokolski M. (2024). Myocarditis Associated with COVID-19 Vaccination. Vaccines.

[155] Buoninfante A., Andeweg A., Genov G., Cavaleri M. (2024). Myocarditis Associated with COVID-19 Vaccination. NPJ Vaccines.

[156] Verbeke R., Lentacker I., De Smedt S.C., Dewitte H. (2025). Three Decades of Messenger RNA Vaccine Development. Nano Today.

[157] Nunez-Castilla J., Stebliankin V., Baral P., Balbin C.A., Sobhan M., Cickovski T., Mondal A.M., Narasimhan G., Chapagain P., Mathee K. (2022). Potential Autoimmunity Resulting from Molecular Mimicry between SARS-CoV-2 Spike and Human Proteins. Viruses.

[158] Arutyunov G.P., Tarlovskaya E.I., Arutyunov A.G., Lopatin Y.M., ACTIV Investigators (2023). Impact of Heart Failure on All-Cause Mortality in COVID-19: Findings from the Eurasian International Registry. ESC Heart Fail..

[159] Heymans S., Cooper L.T. (2022). Myocarditis after COVID-19 mRNA Vaccination: Clinical Observations and Potential Mechanisms. Nat. Rev. Cardiol..

[160] McDonagh T.A., Metra M., Adamo M., Gardner R.S., Baumbach A., Böhm M., Burri H., Butler J., Čelutkienė J., Authors/Task Force Members (2022). 2021 ESC Guidelines for the Diagnosis and Treatment of Acute and Chronic Heart Failure: Developed by the Task Force for the Diagnosis and Treatment of Acute and Chronic Heart Failure of the European Society of Cardiology (ESC). With the Special Contribution of the Heart Failure Association (HFA) of the ESC. Eur. J. Heart Fail..

[161] Heidenreich P.A., Bozkurt B., Aguilar D., Allen L.A., Byun J.J., Colvin M.M., Deswal A., Drazner M.H., Dunlay S.M., Evers L.R. (2022). 2022 AHA/ACC/HFSA Guideline for the Management of Heart Failure: A Report of the American College of Cardiology/American Heart Association Joint Committee on Clinical Practice Guidelines. Circulation.

[162] Jaiswal V., Mukherjee D., Peng Ang S., Kainth T., Naz S., Babu Shrestha A., Agrawal V., Mitra S., Ee Chia J., Jilma B. (2023). COVID-19 Vaccine-Associated Myocarditis: Analysis of the Suspected Cases Reported to the EudraVigilance and a Systematic Review of the Published Literature. Int. J. Cardiol. Heart Vasc..

[163] Myocarditis and Pericarditis After COVID-19 Vaccination: Clinical Management Guidance for Healthcare Professionals. https://www.gov.uk/government/publications/myocarditis-and-pericarditis-after-covid-19-vaccination/myocarditis-and-pericarditis-after-covid-19-vaccination-guidance-for-healthcare-professionals.

[164] Sularz A.K., Hua A., Ismail T. (2023). SARS-CoV-2 Vaccines and Myocarditis. Clin. Med..

[165] Sharma Y.P., Agstam S., Yadav A., Gupta A., Gupta A. (2021). Cardiovascular Manifestations of COVID-19: An Evidence-Based Narrative Review. Indian J. Med. Res..

[166] Kirkpatrick J.N., Swaminathan M., Adedipe A., Garcia-Sayan E., Hung J., Kelly N., Kort S., Nagueh S., Poh K.K., Sarwal A. (2023). American Society of Echocardiography COVID-19 Statement Update: Lessons Learned and Preparation for Future Pandemics. J. Am. Soc. Echocardiogr..

[167] Davis M.G., Bobba A., Chourasia P., Gangu K., Shuja H., Dandachi D., Farooq A., Avula S.R., Shekhar R., Sheikh A.B. (2022). COVID-19 Associated Myocarditis Clinical Outcomes among Hospitalized Patients in the United States: A Propensity Matched Analysis of National Inpatient Sample. Viruses.

[168] Raman B., Bluemke D.A., Lüscher T.F., Neubauer S. (2022). Long COVID: Post-Acute Sequelae of COVID-19 with a Cardiovascular Focus. Eur. Heart J..

[169] Jain S.S., Anderson S.A., Steele J.M., Wilson H.C., Muniz J.C., Soslow J.H., Beroukhim R.S., Maksymiuk V., Jacquemyn X., Frosch O.H. (2024). Cardiac Manifestations and Outcomes of COVID-19 Vaccine-Associated Myocarditis in the Young in the USA: Longitudinal Results from the Myocarditis After COVID Vaccination (MACiV) Multicenter Study. EClinicalMedicine.

[170] Xu Y., Li H., Santosa A., Wettermark B., Fall T., Björk J., Börjesson M., Gisslén M., Nyberg F. (2025). Cardiovascular Events Following Coronavirus Disease 2019 Vaccination in Adults: A Nationwide Swedish Study. Eur. Heart J..

[171] Kaliyaperumal D., Bhargavi K., Ramaraju K., Nair K.S., Ramalingam S., Alagesan M. (2022). Electrocardiographic Changes in COVID-19 Patients: A Hospital-Based Descriptive Study. Indian J. Crit. Care Med..

[172] Moll-Bernardes R., Mattos J.D., Schaustz E.B., Sousa A.S., Ferreira J.R., Tortelly M.B., Pimentel A.M.L., Figueiredo A.C.B.S., Noya-Rabelo M.M., Sales A.R.K. (2022). Troponin in COVID-19: To Measure or Not to Measure? Insights from a Prospective Cohort Study. J. Clin. Med..

[173] Majure D.T., Gruberg L., Saba S.G., Kvasnovsky C., Hirsch J.S., Jauhar R., Northwell Health COVID-19 Research Consortium (2021). Usefulness of Elevated Troponin to Predict Death in Patients with COVID-19 and Myocardial Injury. Am. J. Cardiol..

[174] Smith S.C., Ladenson J.H., Mason J.W., Jaffe A.S. (1997). Elevations of Cardiac Troponin I Associated with Myocarditis. Experimental and Clinical Correlates. Circulation.

[175] Usta Atmaca H., Çiçek N.E., Pişkinpaşa M.E. (2023). Role of Serum NT-proBNP Levels in Early Prediction of Prognosis in Severe COVID-19 Pneumonia. Istanb. Med. J..

[176] Faridi K.F., Hennessey K.C., Shah N., Soufer A., Wang Y., Sugeng L., Agarwal V., Sharma R., Sewanan L.R., Hur D.J. (2020). Left Ventricular Systolic Function and Inpatient Mortality in Patients Hospitalized with Coronavirus Disease 2019 (COVID-19). J. Am. Soc. Echocardiogr..

[177] Schulz-Menger J., Collini V., Gröschel J., Adler Y., Brucato A., Christian V., Ferreira V.M., Gandjbakhch E., Heidecker B., Kerneis M. (2025). 2025 ESC Guidelines for the Management of Myocarditis and Pericarditis. Eur. Heart J..

[178] Overton P.M., Toshner M., Mulligan C., Vora P., Nikkho S., de Backer J., Lavon B.R., Klok F.A. (2022). Pulmonary Thromboembolic Events in COVID-19—A Systematic Literature Review. Pulm. Circ..

[179] Doeblin P., Jahnke C., Schneider M., Al-Tabatabaee S., Goetze C., Weiss K.J., Tanacli R., Faragli A., Witt U., Stehning C. (2022). CMR Findings after COVID-19 and after COVID-19-Vaccination—Same but Different?. Int. J. Cardiovasc. Imaging.

[180] Ferreira V.M., Schulz-Menger J., Holmvang G., Kramer C.M., Carbone I., Sechtem U., Kindermann I., Gutberlet M., Cooper L.T., Liu P. (2018). Cardiovascular Magnetic Resonance in Nonischemic Myocardial Inflammation: Expert Recommendations. J. Am. Coll. Cardiol..

[181] Mahrholdt H., Wagner A., Deluigi C.C., Kispert E., Hager S., Meinhardt G., Vogelsberg H., Fritz P., Dippon J., Bock C.-T. (2006). Presentation, Patterns of Myocardial Damage, and Clinical Course of Viral Myocarditis. Circulation.

[182] Li D.L., Davogustto G., Soslow J.H., Wassenaar J.W., Parikh A.P., Chew J.D., Dendy J.M., George-Durrett K.M., Parra D.A., Clark D.E. (2022). Characteristics of COVID-19 Myocarditis with and Without Multisystem Inflammatory Syndrome. Am. J. Cardiol..

[183] Li Z., Qin L., Xu X., Chen R., Zhang G., Wang B., Li B., Chu X.-M. (2025). Immune Modulation: The Key to Combat SARS-CoV-2 Induced Myocardial Injury. Front. Immunol..

[184] Abumayyaleh M., Schupp T., Behnes M., El-Battrawy I., Hamdani N., Akin I. (2025). COVID-19 and Myocarditis: Trends, Clinical Characteristics, and Future Directions. J. Clin. Med..

[185] Su Y., Chen D., Yuan D., Lausted C., Choi J., Dai C.L., Voillet V., Duvvuri V.R., Scherler K., Troisch P. (2020). Multi-Omics Resolves a Sharp Disease-State Shift between Mild and Moderate COVID-19. Cell.

[186] Sabit H., Arneth B., Altrawy A., Ghazy A., Abdelazeem R.M., Adel A., Abdel-Ghany S., Alqosaibi A.I., Deloukas P., Taghiyev Z.T. (2025). Genetic and Epigenetic Intersections in COVID-19-Associated Cardiovascular Disease: Emerging Insights and Future Directions. Biomedicines.

[187] Xu S., Ilyas I., Weng J. (2023). Endothelial Dysfunction in COVID-19: An Overview of Evidence, Biomarkers, Mechanisms and Potential Therapies. Acta Pharmacol. Sin..

